# Mapping cancer dynamics from normal tissue to malignancy using N- and T-gene expression markers

**DOI:** 10.3389/fsysb.2026.1845582

**Published:** 2026-07-22

**Authors:** Gabriel Gil, Rolando Perez, Augusto Gonzalez

**Affiliations:** 1 Institute of Cybernetics, Mathematics and Physics, Havana, Cuba; 2 Center for Molecular Immunology, Havana, Cuba

**Keywords:** carcinogenesis, N- and T-genes, prostate adenocarcinoma, somatic evolution, systems biology, tumor progression

## Abstract

**Background:**

Carcinogenesis involves two major phases: somatic evolution of normal tissue toward tumor formation, followed by tumor progression toward malignancy. Standard differential expression analysis cannot assign genes specifically to either phase. We introduce N- and T-genes with exclusive expression intervals for normal tissue and tumors, respectively, as markers that could potentially map these dynamics.

**Methods:**

Using TCGA RNA-Seq data from prostate adenocarcinoma (PRAD), lung adenocarcinoma (LUAD) and liver hepatocellular carcinoma (LIHC), we identify N- and T-genes through statistically significant expression intervals exclusive to normal or tumor samples, respectively. We discretize expression into three states (*e* = −1, 0, +1) and count the number of active N-genes in normal samples and active T-genes in tumor samples. Under an ergodic assumption, these counts correlates with pseudo-temporal coordinates for somatic evolution and tumor progression, respectively. We construct complete gene panels (100% sensitive and 100% specific within the training set) using a previously defined algorithm, and perform a molecular taxonomy of normal and tumor samples based on these panels.

**Results:**

Across different cancer types, normal and tumor samples occupy two well-separated attractors in gene-expression space, giving rise to large sets of N- and T-genes. The number of active N-genes decreases continuously as samples move away from the normal attractor, whereas the number of active T-genes increases as samples progress towards the tumor attractor. Large blocks of N-genes are observed, suggesting coordinated multi-gene deactivation events. The combination of staging through the number of active genes with the molecular taxonomy based on panel genes provides a complete description of the somatic evolution of a normal tissue and the progression towards malignancy of tumors.

**Conclusion:**

The N- and T-gene framework provides a natural decomposition of carcinogenesis into somatic evolution (loss of N-gene activity) and tumor progression (gain of T-gene activity). Active N- and T-gene counts can be interpreted as tally marks of somatic evolution and tumor progression, respectively, while complete gene panels enable a taxonomy of samples and helps identifying the biological programs active in each sample. The framework is general and applicable across cancer types, although quantitative results depend on the underlying dataset.

## 1 Introduction

### 1.1 The multi-step model of cancer and its gene-level gap

According to the phenomenological multi-step model of cancer, the accumulation of genetic and epigenetic alterations in a normal tissue may ultimately lead to tumor formation (Frank, 2007). Some attempts have been made to formulate multi-step like models of cancer at the gene level, a well-known example of which is the Vogelstein model of colon cancer (Fearon and Vogelstein, 1990). However, no comparably detailed description exists for most cancer types and tissue localizations. In general terms, tumorigenesis is expected to involve the activation of oncogenes and the deactivation of tumor suppressor genes, but little more is known at a mechanistic level. Which specific genes or gene types are involved in the process? How many genes must be activated or deactivated to trigger the transition? These questions still lack a general and definite answer.

### 1.2 Limitations of differential expression analysis for inferring dynamics

Once the tumor is formed, it undergoes clonal evolution (Greaves and Maley, 2012). In conventional differential gene-expression analysis, pre-tumoral somatic deterioration, tumor initiation and tumor progression are treated as a single event. As a result, genes ranked solely by their differential expression values are not associated specifically with any of these processes. Consider, for example, the *EPHA10* gene in prostate adenocarcinoma (PRAD). In Figure 2a, we show expression histograms, constructed from TCGA data (Tomczak et al., 2015; The Cancer Genome Atlas Research Network, 2015), in normal and tumor samples. The gene is overexpressed in tumors and may therefore be a case of an oncogene. But when could it play a role: in the process of tumor formation or in tumor evolution thereafter? This ambiguity motivates the need for a marker system that can distinguish between these two phases.

### 1.3 The N- and T-gene concept: exclusive expression intervals as dynamic probes

In the present paper, we develop a new paradigm of gene expression analysis based on specific classes of tissue markers (Gil et al., 2025). These markers, namely N-genes and T-genes, exhibit expression intervals that are exclusive to normal or tumor samples, respectively, and thus lend themselves as tools to map the transition from homeostasis to cancer. *EPHA10* is an example of a T-gene whose tumor-exclusive interval lies above the normal range. Like other members of its class, it should play a role in the tumor progression phase. Indeed, the expression of this gene does not vary significantly during pre-tumoral stages. However, when or after the tumor is formed, *EPHA10* may reach high expression values, thereby acting as a marker of progression.

### 1.4 Coarse- and fine-grained dynamics in cancer

The present manuscript focuses on phenomenological trends in the number of active markers associated with the evolution of the normal and tumor tissues. Indeed, changes in the number of active markers may be understood as a coarse-grained dynamics of cancer. A companion paper (Castel et al., 2026) builds upon this foundation by constructing explicit deregulation networks (GDNs) from the same activation data to simulate the spontaneous evolution of normal and tumor tissue. This constitutes a fine-grained representation of the same dynamics. Thus, the present paper provides the conceptual and statistical precedent, while Ref. (Castel et al., 2026) provides the more detailed, network-based, and mechanistic description of the underlying dynamics. Both papers are self-contained but complementary.

### 1.5 Structure of this paper

**FIGURE 1:**
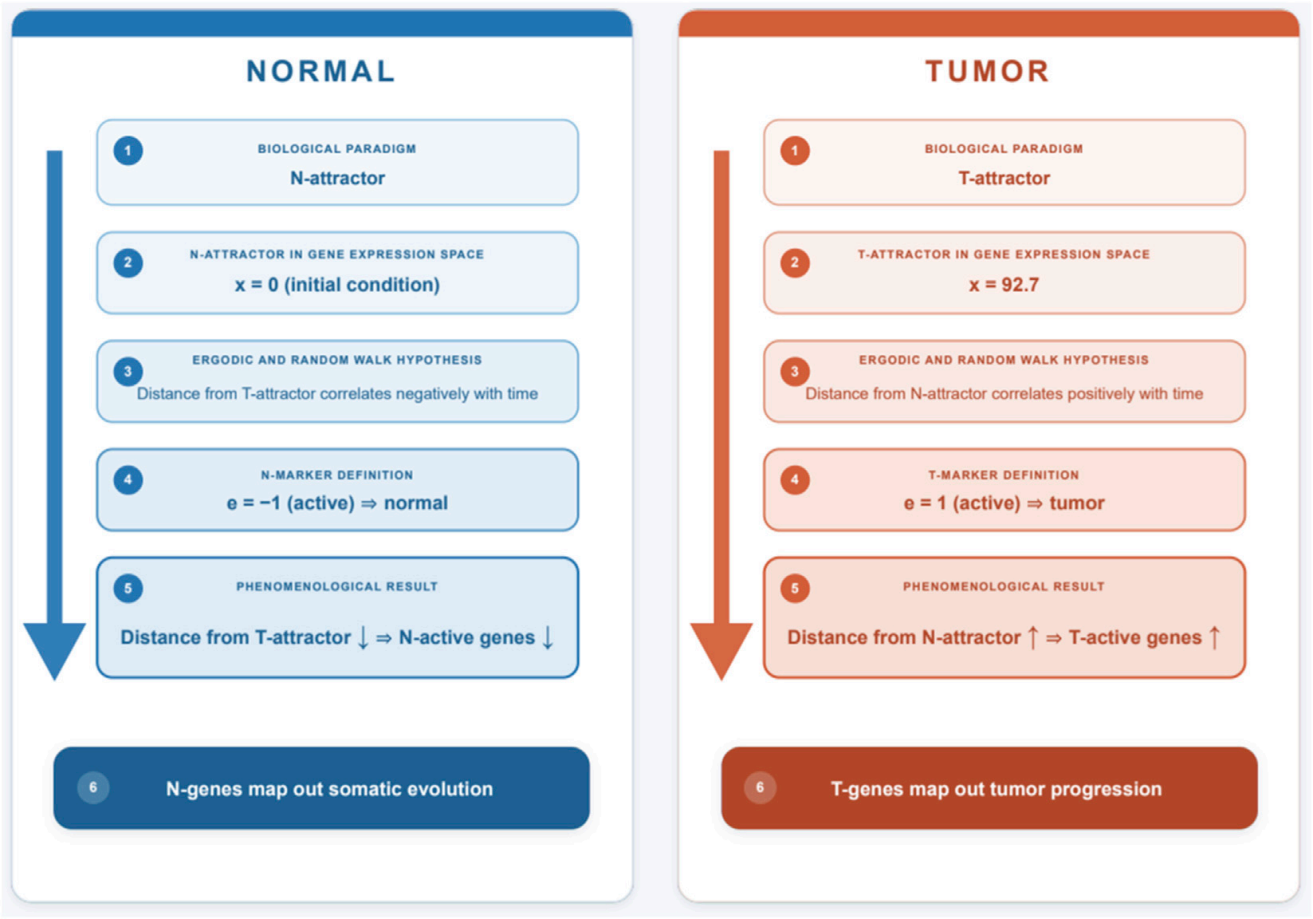
Conceptual diagram of the N- and T-gene framework for mapping cancer dynamics. The diagram summarizes six key components of the framework. (1) Biological paradigm: normal and tumor tissues correspond to stable N- and T-attractors, respectively, following the Kauffman view of gene-regulatory networks. (2) Representation in gene-expression space: normal and tumor samples occupy distinct regions whose centroids approximate the N- and T-attractors. In the PRAD dataset shown here, their coordinates are represented as *x* = 0 and *x* = 92.7, respectively. (3) Ergodic interpretation and random-walk hypothesis: cross-sectional cohorts sample states visited along a stochastic dynamical trajectory. Distance of a normal sample from the T-attractor correlates negatively with time, whereas distance of a tumor sample from the N-attractor correlates positively with time. (4) Marker definition: expression is discretized as *e* = −1, 0, 1; active N-genes are defined by *e* = −1, whereas active T-genes are defined by *e* = 1. (5) Phenomenological result: N-gene activity decreases as the distance from the T-attractor decreases, whereas T-gene activity increases with distance from the N-attractor. (6) Interpretation: N-genes map out somatic tissue evolution, whereas T-genes map out tumor progression.

The logical structure of the paper, its main assumptions and results, is schematically represented in Figure 1. The Methods section describes data acquisition from TCGA, the statistical definition of N- and T-genes, the discretization of expression states, the interpretation of cross-sectional data in terms of ergodicity and stochastic motion hypotheses, and the complete (100% sensitive and 100% specific) gene panels that classify samples. Section 3 (Results and Discussion) is organized as follows. First, we show that the number of active N-genes in normal samples decreases continuously with somatic evolution, while active T-genes increase with tumor progression. Next, we conceptualize the transition between attractors, noting the potential role of NT-genes within it. We then build an 11 N-gene panel that provides a perfect molecular taxonomy of normal samples, and an 8 T-gene panel that provides a perfect taxonomy of tumors. The molecular taxonomy is combined with staging through the number of active genes in order to get a complete description of the somatic evolution of a normal tissue and the progression of tumors. Section 4 (Discussion) summarizes contributions, relates to the companion paper (Castel et al., 2026), and includes a dedicated limitations subsection. The paper ends with concluding remarks.

## 2 Methods

### 2.1 Data acquisition and workflow

Gene expression data for normal tissue samples and tumors were obtained from The Cancer Genome Atlas public network (TCGA, https://portal.gdc.cancer.gov/) (Tomczak et al., 2015). We focus on prostate adenocarcinoma (PRAD) (The Cancer Genome Atlas Research Network, 2015), lung adenocarcinoma (LUAD) (The Cancer Genome Atlas Research Network, 2014), and liver hepatocarcinoma (LIHC) (The Cancer Genome Atlas Research Network, 2017) to illustrate how N- and T- genes are useful for mapping cancer dynamics.

The choice of conventional bulk RNA-seq data instead of single-cell data (Haque et al., 2017) obeys pragmatic reasons. The N- and T-classification requires the detection of significantly populated normal- and tumor-exclusive intervals, which is currently more reliable in the large cohorts typically available from bulk RNA-seq studies. Such data naturally confound pure gene-expression variability with changes in cellular composition. However, in this study, we are interested in evaluating the net effect of these contributions on the overall gene-expression signal, without attempting to disentangle them.

Our framework proceeds in three stages. First, genes are classified into N- and T-gene categories according to the existence of sufficiently populated expression intervals that are exclusive to normal or tumor samples (Gil et al., 2025). Second, the expression of marker genes is discretized into activation states according to whether the expression value falls in a normal-exclusive interval, a tumor-exclusive interval, or a common interval. Third, using the algorithm introduced in Ref. (Gil et al., 2025), we construct full-coverage N- and T-gene panels that achieve 100% sensitivity and 100% specificity in the training samples. Gene classification and panel construction were performed using the in-house code GenePan available at https://github.com/gabriel-gil/GenePan.

### 2.2 N-genes, T-genes and O-genes: a geometric motivation

Normal and tumor samples occupy distinct regions of gene-expression space, commonly referred to as the N and T attractors (Huang et al., 2009; Gonzalez et al., 2021; Gonzalez et al., 2023). Using log-fold expression values for the 60,000 recorded genes (protein-coding and RNA genes) in TCGA data (Gonzalez et al., 2021; Gonzalez et al., 2023), we obtain an Euclidean distance between the attractor centers of approximately 92.7 units in PRAD (Gonzalez et al., 2021; Nieves and Gonzalez, 2024). Let *x* denote the coordinate along the axis joining the centers of the N and T attractors. Normal samples are concentrated around *x* = 0, whereas tumor samples cluster around *x* = 92.7. Additionally, the N and T manifolds are roughly orthogonal to the *x* axis (Nieves and Gonzalez, 2024). The *x* coordinate is a metagene (Huang et al., 2003), receiving contributions from many genes. Some of the genes contributing to *x* are expected to exhibit expression intervals characteristic of the normal state or the tumor. These genes are termed N-genes and T-genes, respectively. However, the full expression range of genes contributing to directions orthogonal to *x* is expected to be accessible to both normal samples and tumors. These genes are referred to as O-genes (O for others). Genes that have both N-only and T-only intervals are called NT-genes.

### 2.3 Examples of N-genes, T-genes and NT-genes

**FIGURE 2:**
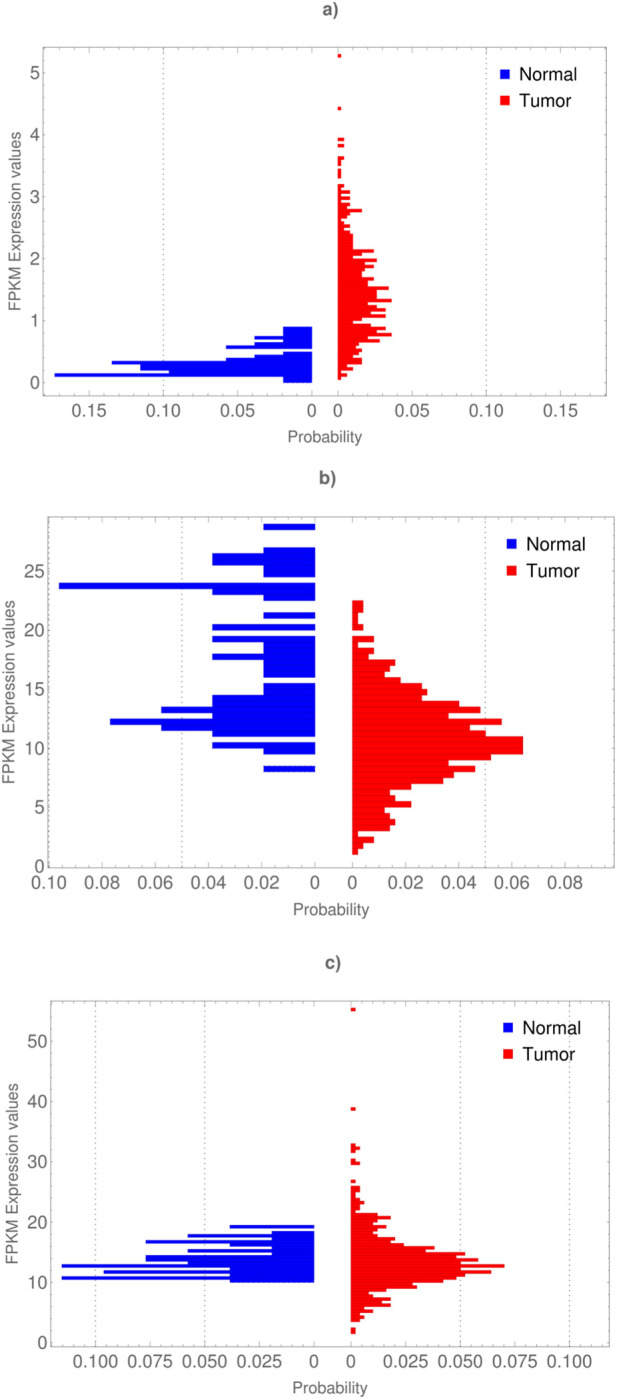
Expression histograms illustrating N-, T- and NT-gene definitions in PRAD. Paired histograms of FPKM expression values for normal samples (blue) and tumor samples (red). **(a)** EPHA10: a T-gene with a tumor-exclusive interval above the normal range (tumor-above). **(b)** SEPTIN10: an NT-gene that shows both a normal-exclusive interval (above ∼ 22.26, normal-above) and a tumor-exclusive interval (below ∼ 8, tumor-below). **(c)** PCMTD1: a T-outside gene with tumor-exclusive intervals both above and below the common region.

The existence of N- and T-genes can be verified directly from the expression data. Consider the paired (normal - tumor) expression histograms for *EPHA10* in PRAD (Figure 2a). FPKM expression intervals for normal and tumor samples are (0.04, 0.88) and (0.08, 5.25), respectively. The interval (0.88, 5.25) is exclusive to tumor samples. EPHA10 is consequently classified as a T-gene (tumor-above).

Consider *SEPTIN10* as a second example (Figure 2b). Expression values above 22.26 are exclusively populated by normal samples, so it is an N-gene (normal-above). However, expression values below 8 FPKM are observed only in tumor samples, making it also a T-gene (tumor-below). This double condition makes *SEPTIN10* an NT-gene. Not all NT-genes are tumor-below/normal-above; for instance, in LUAD, *AGER* behaves as an NT-gene with normal-below and tumor-above exclusive intervals.

Normal-below, normal-above, tumor-below, and tumor-above genes constitute only four subclasses. Consider *PCMTD1* (Figure 2c). There are expression intervals exclusive to tumor samples both above and below the normal region, making *PCMTD1* a tumor-outside T-gene. Likewise, normal-outside N-genes exist (e.g., *LYPD3*). These genes illustrate a feature not captured by conventional differential expression: since their average expression is similar in normals and tumors, they are unlikely to be detected by methods relying on mean shifts. Notably, *PCMTD1* is a fusion gene in PRAD (Yu et al., 2023), and *LYPD3* encodes a highly glycosylated cell surface protein expressed in different types of cancers. Their identification highlights the ability of our framework to detect biologically relevant genes through the structure of expression intervals.

### 2.4 Significance screening of N- and T-genes

To avoid classifying genes on the basis of spurious intervals arising from finite sample sizes, we require each exclusive interval to contain a minimum number of samples determined by an exact Fisher test (Hoffman, 2019). Let *n* be the number of samples in a normal-exclusive or tumor-exclusive interval. Requiring that such an interval does not arise by chance (*p*-value = 0.01) yields:

$$n\;>2\ln\left(10\right)/\ln\left[\left(N+T\right)/N\right]$$

$$n\;>2\ln\left(10\right)/\ln\left[\left(N+T\right)/T\right]$$

for N-genes and T-genes, respectively, where *N* and *T* are the numbers of normal and tumor samples. For the TCGA PRAD cohort (*N* ≈ 50, *T* ≈ 500), this gives *n/N* > 4% and *n/T* > 9%. We adopt slightly stricter thresholds: 5% for N-genes and 10% for T-genes.

Applying these criteria to PRAD yields 1097 N-genes and 6138 T-genes, of which 316 belong to the NT category. Thus, approximately 7,200 marker genes are identified, a substantial reduction from ∼ 60,000 recorded genes in the TCGA database (protein-coding and RNA genes). The remaining genes are O-genes or not expressed in the tissue. The larger number of T-genes reflects the higher transcriptional disorder of tumors (Mesa-Rodriguez et al., 2022; Gonzalez et al., 2022), as previously discussed in Ref. (Gil et al., 2025).

### 2.5 Expression states, N- and T-activity

We discretize continuous gene expression into three biologically meaningful states:

- *e* = −1 (N-active), when the expression lies in a normal-exclusive interval.
- *e* = 1 (T-active), when the expression lies in a tumor-exclusive interval.
- *e* = 0, otherwise (common interval or non-significant exclusive intervals).

For T-genes such as *EPHA10*, *e* = 1 is specific to tumor samples, serving as a tumor marker. For N-genes, *e* = −1 is specific to normal samples, serving as a normal marker. NT-genes such as *SEPTIN10* may exhibit both N-marker and T-marker behavior, depending on the expression interval occupied by the sample. The exclusive intervals represent expression states incompatible with the complementary phenotype. Therefore, if *SEPTIN10* is N-active in a normal sample, the transition toward the tumor phenotype requires the loss of its N-active state (change from *e* = −1 to *e* = 0). During the transition or after tumor formation, it may be activated as a T-marker (further change from 0 to 1).

### 2.6 Dynamical interpretation of cross-sectional data: attractors, ergodicity and stochastic trajectories

As introduced in Section 2.2, normal and tumor samples occupy distinct, well-separated regions in gene-expression space, which we refer to as the N-attractor and T-attractor, respectively (Huang et al., 2009; Gonzalez et al., 2021; Gonzalez et al., 2023). These attractors are interpreted as stable biological states corresponding to local maxima of a fitness landscape. Each attractor comprises a set of accessible expression states, whose empirical distribution can be approximated from cross-sectional, multi-patient RNA-seq data (Gonzalez et al., 2022).

To extract dynamical information from these static measurements, we adopt an ergodic assumption (Cornfeld et al., 2012). Under this assumption, the ensemble of samples collected from different individuals at given time points is considered to sample the set of states that a single tissue would visit along its evolutionary trajectory. Thus, cross-sectional data inform not only on the equilibrium properties of the attractors but also on the statistical structure of the transition dynamics between them.

We further describe carcinogenesis as a stochastic trajectory in gene-expression space. Starting from a normal state within the basin of the N-attractor, the tissue undergoes sequential perturbations (somatic mutations, epigenetic alterations, etc.) that cause it to move randomly in expression space. Over time, the expected squared displacement from the initial condition increases, akin to a random walk (Herrero et al., 2022). Consequently, states that lie farther from the N-attractor are interpreted, on average, as corresponding to later stages of pre-tumoral evolution. Conversely, for tumor samples, larger distances from the N-attractor (or equivalently, deeper entry into the T-attractor basin) indicate more advanced tumor progression.

Together, the stochastic evolution and ergodic assumptions provide a formal justification for using the Euclidean distance from a sample to the N-attractor (or T-attractor) as a proxy for its relative stage along the normal-to-malignant continuum. This distance-based ordering is used throughout the Results section to correlate with the number of active N- or T-markers, without implying a deterministic time scale. The assumptions and their limitations are further discussed in Section 4.3.

### 2.7 Construction of complete (exhaustive) gene panels

We construct “complete” panels (100% sensitivity and 100% specificity within the training set) using an algorithm described in Ref. (Gil et al., 2025). For the N-panel, we start with the N-gene that has the highest frequency of activation (i.e., proportion of normal samples where *e* = −1). We then iteratively add the gene that covers the largest number of yet-uncovered normal samples (i.e., samples where no panel gene is yet active). The process stops when all normal samples have at least one active panel gene. The resulting set is minimal in the sense that removing any gene would break completeness (or full coverage). For the T-panel, the same algorithm is applied to tumor samples using T-activation (*e* = 1). The full coverage of the N- and T-panels is dataset-specific. However, in Gil et al. (2025) we show that the number of genes in a panel saturates for large enough sample sets.

### 2.8 Stability tests

Subsampling stability are described in detail in (Castel et al., 2026). Here, we briefly summarize the results for self-consistency. For PRAD, 100 replicates of random subsamples comprising all 52 normal samples and varying numbers of tumor samples were generated to assess recall and precision of T-gene identification. To evaluate the stability of the N-gene set and its associated block structure, we also performed analogous analysis varying the number of normal samples while keeping the full tumor cohort (499 samples) fixed.

These analyses showed that a large fraction of the detected N- and T-genes (> 80%) was preserved across subsamples. Likewise, the overall organization of the N-gene blocks remained stable, as reflected by the weak dependence of the largest block size on the number of normal samples. The results therefore indicate that the observed patterns are robust and do not arise as artifacts of the specific frequency thresholds used for gene classification.

## 3 Results and discussion

### 3.1 N-markers and the somatic evolution of a normal sample

We first argue that, in the spontaneous somatic evolution of a normal sample, only the N-markers are relevant. Indeed, consider a discrete expression model to describe the dynamics, as in Ref. (Harris et al., 2002). In this model, the O-genes always take the value *e* = 0 and do not participate in the dynamics. On the other hand, for T-genes, *e* = 1 already marks a tumor state; therefore, none of the T-genes can be activated during the pre-tumoral dynamics. This leaves us with the N-marker dynamics as effective description of the initial somatic evolution.

In differential expression analysis, normal samples are used only to set reference values for gene expression. However, normal samples are ideal for the study of carcinogenesis, since they represent normal tissues at different stages of somatic evolution.

**FIGURE 3:**
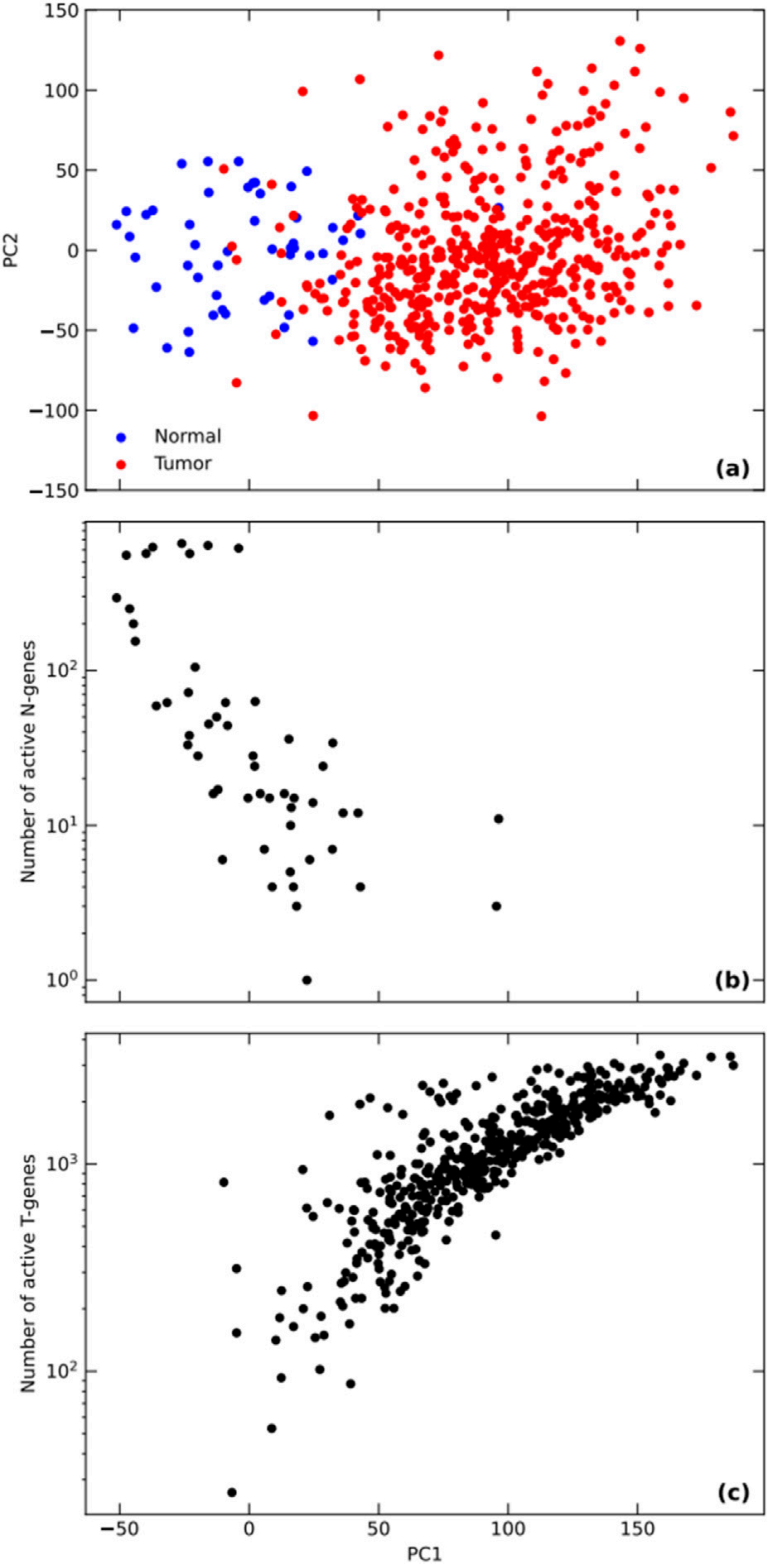
Principal component analysis (PCA) of PRAD samples and active N/T gene counts. **(a)** PCA projection of normal (blue) and tumor (red) samples onto the first two principal components. PC1 separates normal from tumor attractors (centers at x = 0 and x = 92.7, respectively). **(b)** Number of active N-genes in each normal sample plotted against PC1 coordinate. The number decreases from ∼ 600 (healthy-like) to ∼ 1 (near transition). **(c)** Number of active T-genes in each tumor sample plotted against PC1 coordinate. The number increases from ∼ 20 (recently formed tumors) to >3,000 (advanced tumors). PC1 captures the main axis of transcriptional change from normal to malignant states.

We show in Figure 3a a reduced-dimension representation of PRAD data. In particular, Figure 3a shows clouds of normal and tumor samples as obtained from Principal Component Analysis (PCA) performed along the lines of Ref. (Gonzalez et al., 2023). The center of the normal cloud of samples is at *x* = 0 (PC1 coordinate), whereas the center of the tumor cloud is at *x* = 92.7.

In Figure 3b we use the same PC1 axis, but the ordinate is the number of active N-markers in the 52 normal samples within the dataset. Notice that this number ranges from 1 to about 600. Samples closer to the center of the normal attractor exhibit healthier conditions associated with larger numbers of active N-genes (500 or more), whereas this number decreases during somatic evolution as the samples approach the transition region. It is remarkable that a single active N-gene has the potential to maintain the normal-state condition, even after more than 500 genes have been deactivated.

**FIGURE 4:**
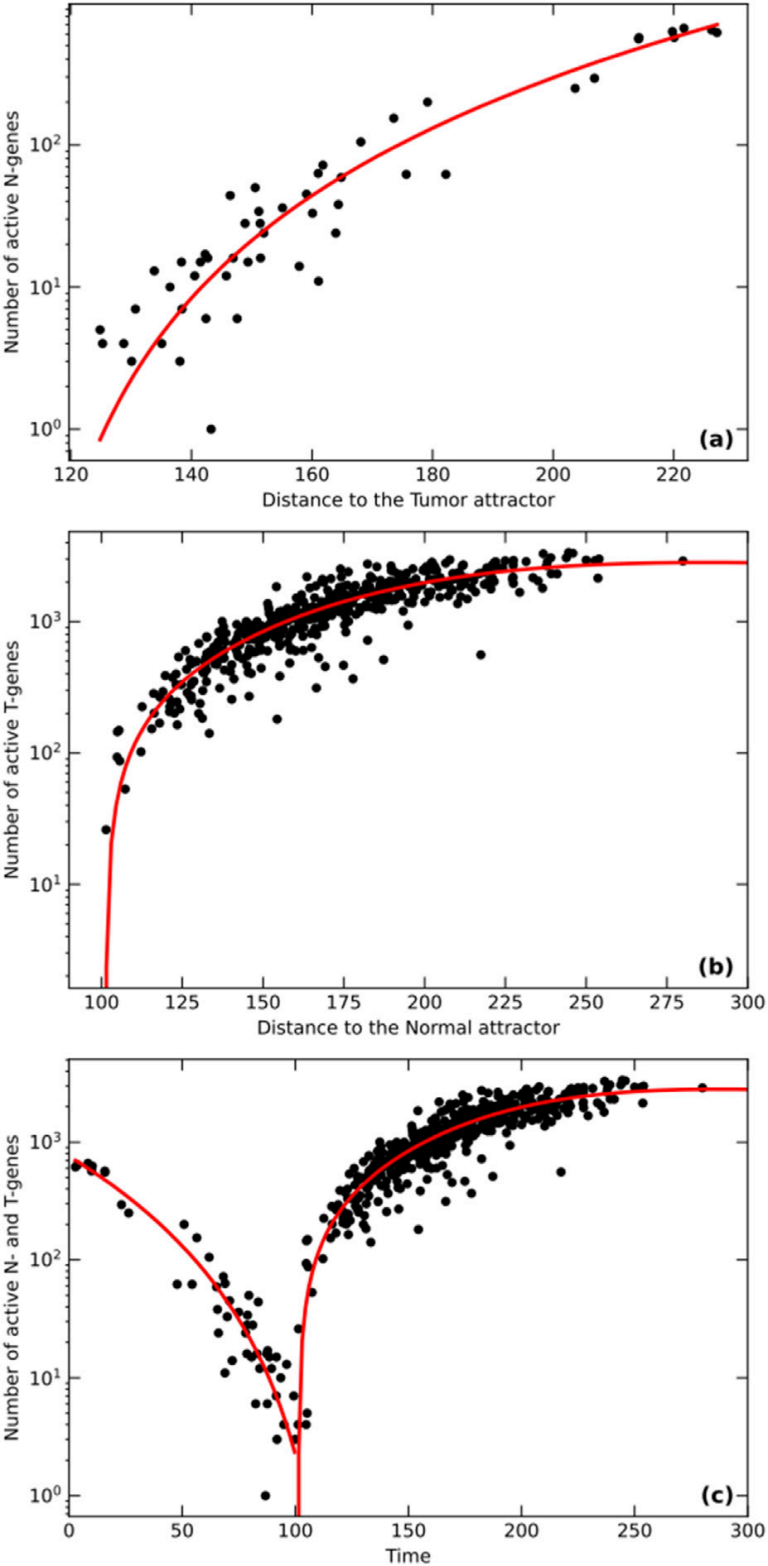
Active N/T gene counts as a function of Euclidean distance to the opposite attractor and schematic transition. **(a)** Active N-genes in normal samples vs. Euclidean distance to the tumor attractor center. A continuous decline is observed, approaching zero near the transition (distance ∼ 120–130 units). **(b)** Active T-genes in tumor samples vs. Euclidean distance to the normal attractor center. A continuous increase is observed, starting from near zero at the transition (distance ∼ 95–105 units). **(c)** Schematic representation of the transition: somatic evolution (left) is characterized by decreasing active N-genes; the transition region (centre) may involve coexistence of N- and T-markers; tumor progression (right) is characterized by increasing active T-genes. Pseudotime is constructed from distances under the ergodic assumption and a rate of one unit of log-fold expression per unit time (for simplicity). The solid lines in **(a–c)** are guides to the eye.

We know that distances are not faithfully represented in PCA diagrams. To eliminate this distortion, Figure 4a uses as the abscissa the actual Euclidean distance between each normal sample and the center of the tumor attractor in the full gene-expression space. The inverse relationship between distance and the number of active N-genes becomes even more apparent. The solid line is included only as a guide to the eye. Nevertheless, the trend is clear: the number of active N-genes progressively decreases toward zero as samples get closer to the tumor attractor.

By combining a cross-sectional description of gene-expression space with the ergodic and stochastic-dynamics assumptions introduced in Section 2.7, we may interpret Figures 3b, 4a as mapping the somatic evolution of normal samples in terms of N-markers. In particular, the number of active N-markers decreases continuously as the normal sample moves away from the N-attractor and toward the T-attractor. We shall use the similar dynamic interpretations hereafter.

To support the ergodic assumption, we note that our PRAD cohort covers a wide range of somatic evolution and tumor progression stages. The continuous gradient in active N- and T-gene counts (Figures 4a,b) without discrete clustering is consistent with the ergodic approximation.

We use the number of active N-genes, rather than the number of deactivated ones, to establish a correlation with the stage of pre-tumoral evolution. This choice is dictated by the nature of cross-sectional RNA-seq data, which provide a single snapshot of each tissue sample. Consequently, the data reveal the N-markers that remain active at the time of measurement, but not those that were deactivated during earlier stages of somatic evolution. Information about the original set of active N-markers is therefore lost along the evolutionary trajectory.

The deactivation dynamics in lung adenocarcinoma [LUAD (The Cancer Genome Atlas Research Network, 2014)] and liver hepatocellular carcinoma [LIHC (The Cancer Genome Atlas Research Network, 2017)] is discussed in Supplementary Section S1 of Supplementary Material. In particular, Supplementary Figures 1a,b show results similar to Figure 3a. The numbers of N-genes in these tissues are 492 and 581, respectively. The slopes of decline are comparable to PRAD, suggesting a conserved deactivation kinetics.

### 3.2 T-markers and the progression of tumors

In the same way, we argue that only T-markers are relevant in the spontaneous dynamics of established tumors, and we use them as tally marks of this process. To this end, we plot in Figure 3c the number of activated T-genes as a function of the PC1 coordinate. This number ranges from 26 to 3,362 in PRAD. The figure shows tumor samples exhibiting very different stages of progression. Samples with relatively few activated T-genes are closer to the normal attractor and likely correspond to recently formed tumors, whereas more progressed tumors exhibit higher numbers of activated T-genes.

Similar to the case for normal samples, we also correlate the actual distance from each tumor sample to the center of the normal attractor with the number of activated T-markers in that sample, as shown in Figure 4b. The tendency is toward zero activated T-genes at the transition.

It is worth noting, however, that the correlation between position along the axis joining the two attractors and the clinically assigned tumor stage is not particularly strong in PRAD. This is likely a consequence of the substantial heterogeneity of this tumor type. In less heterogeneous cancer types, a much clearer relationship is observed, as illustrated, for example, in Figure 5 of Ref. (Gonzalez et al., 2023).

**FIGURE 5:**
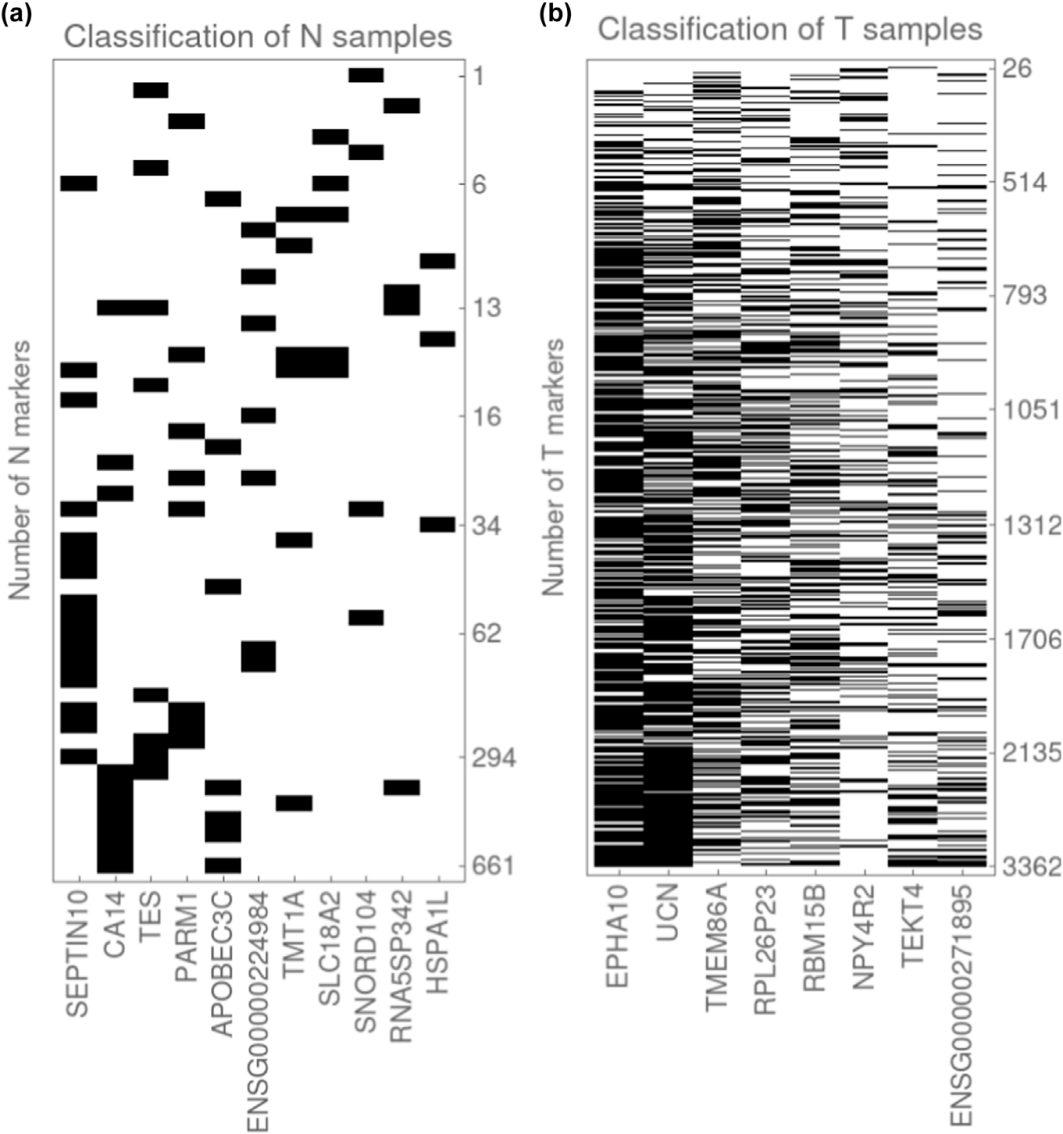
Complete gene panel activity matrices for normal and tumor samples. **(a)** N-panel (11 genes) activity in normal samples. Rows: genes (ordered as in Table 1). Columns: normal samples (ordered from bottom to top by decreasing total active N-genes, i.e., less evolved at the bottom, closer to transition at the top). Black cells indicate active N-gene (e = −1). Every normal sample has at least one active panel gene; most have 1 - 3. **(b)** T-panel (8 genes) activity in tumor samples. Columns: tumor samples (ordered from top to bottom by increasing total active T-genes, i.e., early tumors at the top, advanced at the bottom). Black cells indicate active T-gene (e = 1). Every tumor sample has at least one active panel gene; early tumors typically have 1 - 2, advanced tumors up to 6.

Details of the activation dynamics are also presented in Supplementary Section S1 of the Supplementary Material. Figures 2a,b show similar results for LUAD and LIHC, respectively. The numbers of T-genes in these tumors are 17,037 and 17,351, respectively. The increase in active T-genes with distance from the normal attractor is steeper in LUAD and LIHC than in PRAD, consistent with the larger attractor distances and more aggressive clinical behavior.

In summary, Figures 3c, 4b show that tumor progression is characterized by a continuous increase in the number of T-markers as the sample moves away from the transition region and approaches the central region of the T-attractor. As in the case of normal samples, RNA-seq measurement captures all of the T-markers that are active at a given time. We assume that the observed markers were activated in the interval between transition and the given time, and remain activated thereafter.

### 3.3 Conceptualizing the transition

Recall the diagram in Figure 3a. The normal and tumor attractors are separated by a low-fitness barrier (Gonzalez et al., 2021), resulting in a discontinuous transition between attractors in line with statistical physics (Herrero et al., 2022; Binder, 1987). We can schematically picture the transition as follows: the tissue sample initially lies near the center of the normal attractor (a healthy state). Somatic evolution consists of small, random jumps within the basin until it reaches a point near the boundary. This is followed by an abrupt jump toward the tumor basin (Herrero et al., 2022), after which small, random jumps within the tumor basin take place, representing progression.

In terms of N- and T- genes, first the N-genes anchoring the normal state should be gradually inactivated. When most are down, T-marker activation leads to the transition. A schematic picture is presented in Figure 4c. The x-axis in that figure is constructed from the distances in 4a and 4b, transformed into time using the ergodic principle and a rate of one unit of log-fold expression per unit time (for simplicity). The transition is represented as a point, although it could also be an interval or region where N-markers and T-markers coexist, defining a pre-neoplastic stage, e.g. prostatic intraepithelial neoplasia (PIN). To describe the dynamics at the transition point or region, where there is a shift from predominantly N-marker activity to predominantly T-marker activity, we must include both N- and T-genes and their deregulation networks (Castel et al., 2026).

The number of active N-genes in the normal sample is reduced as a result of perturbations, coming from somatic mutations (Martincorena and Campbell, 2015), epigenetic alterations (Ilango et al., 2020), or external influences (Boffetta and Nyberg, 2003). They may provoke deregulation cascades (Harris et al., 2002) in the regulation network.

Assume the system is in a normal state and a perturbation occurs. The attractor is a stable state and may support small perturbations. If the cascade reaches a subset of the active N-genes, it may shift their expression into the interval common to N and T samples - that is, reducing the expression to *e* = 0. The affected genes then cease to sustain the normal state, effectively taking a step toward tumor formation. In the process, part of the information about the initial normal state (the initially active N-genes) is lost. Notice that a gene cannot be T-activated as long as N-genes remain active, maintaining the normal state.

Thus, the first cascades prepare the transition by inactivating most of the N-genes. However, there should be a cascade that actually realizes the transition, inactivating the remaining N-genes while activating the T-genes that mark the new tumor. Near the transition point, both N- and T-deregulation networks are crucial, with NT-genes potentially bridging the two by switching from N- to T-activity.

The idea of cascades in carcinogenesis is also supported by gene expression data. Data reveal blocks of N-genes that share identical discrete expression profiles, with all genes in a block simultaneously active in the same normal samples. The largest block in PRAD contains 216 genes. Thus, during the somatic evolution of the normal tissue, a single event may trigger the inactivation of all 216 genes at once. This situation recalls the multistep model of cancer (Frank, 2007). We present in Supplementary Section S2 of Supplementary Material, in particular in Supplementary Table 1, more details on block genes in PRAD. The frequency and number of genes for some of these blocks are explicitly given.

Gene blocks could be also consequence of the smaller number of normal samples in the data. However, stability tests and their presence in different tissues points to a general transcriptional characteristic of the normal state (Castel et al., 2026).

### 3.4 Staging and molecular taxonomy of normal tissue using N-genes

In Section 3.1, Figure 4a, we have shown that the stage of somatic evolution of a normal sample can be assessed continuously by the total number of active N-genes it retains: the lower this number, the further the tissue has progressed toward the transition to tumor. We now introduce a qualitative complement: a molecular taxonomy of normal tissue based on complete N-gene panels. These panels, defined in Gil et al. (2025) and summarized in Methods Section 2.7, reveal which protective mechanisms or normal-state anchoring pathways remain active in each sample. Thus, combining the total number of active N-genes (quantitative stage) with the activation pattern of panel genes (qualitative taxonomy) provides a richer characterization of somatic evolution. We illustrate this approach using PRAD data.

We start from the gene with the highest activity frequency in the set of normal samples, *SEPTIN10*, and add genes until all samples in the set show at least one panel gene active. The procedure is detailed in Ref. 6. One realization of this procedure leads to the following panel of 11 genes: *SEPTIN10, CA14, TES, PARM1, APOBEC3C, ENSG00000224984, TMT1A, SLC18A2, SNORD104, RNA5SP342, HSPA1L*. Note that all of them, except SNORD104, belong to the N-above category, i.e., they are tumor suppressor-like genes. In addition, five of them are NT genes: SEPTIN10, TES, PARM1, APOBEC3C, and SNORD104. This means they can switch their role from anchoring in the normal state to boosting tumor progression.

The categories, frequencies and known functional roles for these genes are summarized in Table 1. The latter is a qualitative inference, following from the participation in pathways of the curated Reactome database (Gillespie et al., 2021) and cites from recent literature. The interpretation is limited by current knowledge of gene ontology and functions. Indeed, only 6 of the panel genes are annotated in the Reactome database. Details are shown in Supplementary Section S2 of Supplementary Material, in particular Supplementary Table 2.

**TABLE 1:** Properties of the complete N-genes panel in PRAD.

Perfect panel of N-genes			
Gene symbol	Category	Activation frequency	Known functional role
*SEPTIN10*	NT-gene (N-above, T-below)	0.30	Ensures accurate cell division
*CA14*	N-above	0.19	Reversible hydration of carbon dioxide
*TES*	NT-gene (N-above, T-below)	0.15	Role in surfactant metabolism
*PARM1*	NT-gene (N-above, T-below)	0.15	Androgen-repressed, pro-survival factor
*APOBEC3C*	NT-gene (N-above, T-below)	0.13	Role in mRNA editing
*ENSG00000224984*	N-above	0.13	LncRNA. Uncharacterized
*TMT1A*	N-above	0.11	Role in neutrophil degranulation
*SLC18A2*	N-above, 0.09	0.09	Role in neuronal system
*SNORD104*	NT-gene (N-above, T-below)	0.07	Promote proliferation and migration in prostate cancer
*RNA5SP342*	N-above	0.07	Ribosomal pseudogene. Uncharacterized
*HSPA1L*	N-above	0.05	Role in cellular response to heat stress and in cytokine signaling in immune system

For each gene, the table shows: gene symbol, category (N-above, N-below, NT), frequency of activation in the set of normal samples, and known functional role (condensed). Detailed annotations on the basis of the Reactome database (Gillespie et al., 2021) and recent literature are provided in Supplementary Table 2.

Generally, panel genes do not belong to blocks with more than one member, i.e., they are the sole representatives of a given expression profile. The only exception is HSPA1L, which belongs to a block of four genes with the same expression profile: HSPA1L, HSPA1A, BAG3, DNAJB1, all related to the stress survival network and the stabilization of the androgen receptor.

In Figure 5a we show the classification the panel induces in the set of normal samples. A black cell in Figure 5a indicates that the corresponding gene marker is active. Samples are ordered according to the number of total active N-genes: less evolved samples, with a larger number of N-genes, appear at the bottom, whereas samples closer to tumor formation appear at the top of the figure.

The figure shows a weak correlation between the number of active panel genes and the degree of somatic evolution. Healthy samples typically show two or one, and only rarely three, active genes, whereas evolved samples show, as a rule, only one. Details are shown in Supplementary Section S3 of Supplementary Material. The conclusion is that the number of active genes in the panel is a rough indicator of staging.

The small number of active genes probably suggests that the protection mechanisms are inherited and that only a fraction of all N-genes are active at birth. A second possible explanation is that all men are born with the approximately 1000 N-genes active, but half of them have already become inactive by middle age. More data are needed to test these hypotheses.

We may use the patterns of active panel genes to cluster the samples. This possibility is discussed in Supplementary Section S4 of Supplementary Material. The limited knowledge about panel genes and the dependence on the sample dataset makes its clinical relevance questionable and needed of validation. Nevertheless, we are showing, for the first time, a procedure to construct a complete molecular classification of somatic evolution of the normal tissue based on expression markers. More efforts are needed in this direction, which are beyond the scope of the present paper.

### 3.5 Staging and molecular taxonomy of tumors using T-genes

In Section 3.2; Figure 4b, we have shown that also the stage of tumor progression can be monitored through the total number of active T-genes in a tumor sample. The number of active T-genes increases systematically with progression, providing a quantitative indicator of the tumor’s evolutionary stage. The qualitative complement in this case is a molecular taxonomy of tumors based on complete T-gene panels. The activity of the panel genes indicates which malignancy-promoting pathways or mechanisms are operative in a given tumor. Thus, combining the total number of active T-genes (quantitative stage) with the activation pattern of panel genes (qualitative taxonomy) provides a richer characterization of tumor progression.

Proceeding along the lines of Gil et al. (2025), we obtain the following complete panel of 8 genes: EPHA10, UCN, TMEM86A, RPL26P23, RBM15B, NPY4R2, TEKT4, and ENSG00000271895. All of these genes belong to the T-above group, and therefore display an oncogene-like behavior.

Their properties and qualitative association with functional roles are shown in Table 2. Again, limited knowledge of gene ontology and functions prevents a complete understanding of the role of panel genes in boosting tumors. Only 3 of the panel genes are annotated in the curated Reactome database. Details are shown in Supplementary Section S2 of Supplementary Material, in particular Supplementary Table 2.

**TABLE 2:** Properties of the complete T-genes panel in PRAD.

Perfect panel of T-genes		​	​
Gene symbol	Category	Activation frequency	Known functional role
*EPHA10*	T-above	0.74	Role in nervous system development
*UCN*	T-above	0.64	Role in peptide hormone metabolism and signal transduction
*TMEM86A*	T-above	0.51	Role in phospholipid metabolism
*RPL26P23*	T-above	0.43	Pseudogene (likely). Uncharacterized
*RBM15B*	T-above	0.41	Promotes proliferation
*NPY4R2*	T-above	0.24	Role in neuroendocrine differentiation
*TEKT4*	T-above	0.24	Role in chemotherapy resistance
*ENSG00000271895*	T-above	0.20	Uncharacterized

The same as Table 1 for the T-genes panel. The category of all these genes is T-above (oncogenes-like). Detailed annotations on the basis of the Reactome database (Boffetta and Nyberg, 2003) and recent literature are provided in Supplementary Table 2.

In Figure 5b we show how the use of the number of total active T-genes and active boosting mechanisms, labeled by the panel genes, provides a combined description of tumor samples. Recently formed tumors exhibit one or two active panel genes, whereas progressed tumors show, as a rule, six.

The correlation between the total number of active genes and the number of active genes in the panel is further discussed in Supplementary Section S3 of Supplementary Material. This correlation is much better for tumors than for normal samples, suggesting that the T-panel qualitatively captures the progression axis.

Applying the same clustering algorithm used in the case of normal samples, we get a complete taxonomy of prostate tumors based on expression markers. This is described in Supplementary Section S4 of Supplementary Material. In Supplementary Section S5 we qualitatively compare our classification with one traditional taxonomy based on mutational markers (The Cancer Genome Atlas Research Network, 2015). Of course, all these results are preliminary and deserve much more future work.

## 4 Discussion

### 4.1 Summary of contributions

We have stressed the significance of N- and T- genes in the processes of carcinogenesis and tumor progression. Our conceptualization leads to the following overall picture. The normal tissue starts with a set of active N-markers, which are progressively deactivated during somatic evolution as a result of deregulation cascades. The active N-genes in a given normal sample serve as both markers and anchors of the normal state, and when deactivated, they prime the tissue for a transition toward the tumor. Therefore, the number of active N-markers defines the stage of somatic evolution of the tissue. Moreover, the mechanisms still protecting the tissue may be identified by a small panel of 11 N-genes that completely discriminates between normal and tumor samples within the data.

On the other hand, cascades in established tumors operate on T-markers. Analogously, the number of activated T-markers defines the stage of tumor progression. The mechanisms promoting a more malignant state may be identified by a small panel of 8 T-genes that achieves complete classification of normal and tumor samples within the data.

The 100% sensitivity and 100% specificity of our N- and T-gene panels are sample-dependent, and so is our molecular taxonomy of normal tissue and tumors based on them. Regarding sensitivity and specificity, we have discussed elsewhere (Gil et al., 2025) that some of our panels may be close to complete and are therefore expected to perform accurately in broader or distinct datasets, but this remains to be tested. The same logic applies to the molecular taxonomy. Nonetheless, the classification within either normal tissues or tumors attained by our gene panels requires clinical validation.

### 4.2 Relationship to a companion paper

The present manuscript provides the conceptual and statistical foundation for the N- and T-gene framework. It establishes the existence of exclusive expression intervals, the discretization scheme, the use of active gene counts as temporal correlates, and the use of complete panels for the identification of operative biological programs within each sample type. The companion paper (Castel et al., 2026) builds directly on this foundation by constructing explicit probabilistic deregulation networks (GDNs). We simulate spontaneous evolution and interventions, showing how network topology determines both processes. Thus, the two papers are complementary: the present paper gives the coarse-grained (“thermodynamic”) view, while (Castel et al., 2026) gives the fine-grained network (“kinetic”) view. Both are necessary for a complete understanding of cancer dynamics.

### 4.3 Specific limitations acknowledged in this study

- Bulk RNA-Seq and cellular composition: Our data average over malignant, stromal and immune cells. In addition, surgical resection may include contamination of tumor samples with adjacent normal tissue (or *vice versa*). Our exclusive-interval definition of N- and T-genes with statistical thresholds reduces but does not eliminate the risk of confounding factors. Single-cell RNA-seq will be required to resolve intra-tumor heterogeneity.
- Cross-sectional design and ergodic assumption: We use the ergodic principle to infer dynamics from static snapshots. This assumes that the cohort spans all stages of progression and that different patients’ samples can be aligned along a common trajectory. While our PRAD cohort includes normal samples with varying ages and tumors with varying Gleason scores, and we observe a continuous gradient without discrete clusters, longitudinal or time-series data (e.g., from organoid models or serial biopsies) would provide stronger evidence. The ergodic assumption remains an approximation.
- Threshold sensitivity and sample size dependence: The 5% (N-genes) and 10% (T-genes) thresholds are derived from the Fisher test for the PRAD cohort (N ∼ 52, T ∼ 500). Subsampling analysis (Castel et al. (2026), Section 2.8) shows that with half the tumor bank, approximately 95% of original T-genes are recovered. Nevertheless, thresholds should be re-derived for new datasets, and the exact gene lists are dataset-dependent. Saturation is expected for very large datasets.
- Heterogeneity within tumors: Bulk measurements average over potentially diverse clones. A single tumor may contain subpopulations at different progression stages. Our labels represent the dominant bulk state. Single-cell analysis would be required to map clonal heterogeneity.

### 4.4 Concluding remarks

We have shown that N- and T-genes provide a natural decomposition of carcinogenesis into somatic evolution (loss of N-gene activity) and tumor progression (gain of T-gene activity). Complete gene panels enable a description of the active biological programs for each sample. The framework is general, though quantitative results are dataset-specific. Future work will integrate the coarse-grained dynamics with the network dynamics of (Castel et al., 2026), apply single-cell data to resolve heterogeneity, and test intervention predictions with experimental results.

## Data Availability

All data used in this study are publicly available through the TCGA Research Network (https://www.cancer.gov/tcga). Gene classification and panel construction were performed using the in-house code GenePan available at https://github.com/gabriel-gil/GenePan.
