# Supplementary material for: Mapping cancer dynamics from normal tissue to malignancy using N- and T-gene expression markers
Source: Front Syst Biol. 2026 Jul 22;6:1845582. doi: 10.3389/fsysb.2026.1845582 (PMC13437281; doi:10.3389/fsysb.2026.1845582)
Supplement: Supplementary file 1 [file DataSheet1.pdf]

Mapping cancer dynamics from normal tissue to malignancy using N- and T-gene expression markers

Running title: N- and T-markers in cancer dynamics

Authors: Gabriel Gil[1], Rolando Perez[2], Augusto Gonzalez[1]*

Affiliations:
[1] Institute of Cybernetics, Mathematics and Physics, Havana, Cuba
[2] Center for Molecular Immunology, Havana, Cuba

Correspondence:
Augusto Gonzalez – agonzale@icimaf.cu
Gabriel Gil – gabriel@icimaf.cu

Supplementary Material

S1. Deactivation and activation dynamics in other tumors

For two other tumor localizations, LUAD and LIHC, we show figures analogous to 4a and 4b in order to verify that the number of active markers exhibits a good correlation with distance and, thus, may serve as a quantitative indicator of staging.

Supplementary Figure 1 plots the number of active N-markers as a function of the distance to the T-attractor. As in Figure 4a, red lines are drawn as guides to the eye.

Notably, a rough calculation of slopes for the three tissues yields similar values: PRAD – 600 markers / 100 distance units = 6 markers/unit; LUAD – 200 markers / 40 units = 5 markers/unit; LIHC – 300 markers / 50 units = 6 markers/unit. This result points to a universal deactivation kinetics in normal tissue in the neighborhood of the transition point. Further data are required to corroborate this finding.

On the other hand, Supplementary Figure 2 shows the number of active T-markers as a function of the distance to the N-attractor. The correlation between the number of active markers and distance is again apparent.

However, a rough calculation of slopes yields very different values for the three tumors. This is mainly related to the different numbers of T-genes, which, as noted in Ref. [7], depend on the inter-attractor distance. LUAD and LIHC, with a larger distance, exhibit a larger number of T-genes than PRAD, consistent with the deeper transcriptional reprogramming and more aggressive clinical behavior of these tumors.

S2. Block and panel genes in PRAD (detailed)

There are 492 block genes in the N-genes of PRAD. Supplementary Table I provides the frequencies and sizes of the first 5 blocks. Stability tests reported in section 2.8 of the main paper

and the presence of gene blocks in other tumors [7] indicate that blocks are not merely sample artifacts.

On the other hand, the participation of panel genes in pathways of the curated Reactome database [27], and citations to these genes in recent literature are summarized in Supplementary Table II. This information is the basis for the inferred functional roles of panels genes in Tables I and II of the main text. We stress that such the inference is anyhow qualitative and preliminary due to the limited knowledge on gene ontology and functions.

S3. The number of active panel genes is a rough indicator of progression

In Sections 3.4 and 3.5, we noted that the panel genes not only provide a molecular taxonomy of the tissue but also exhibit a correlation with the total number of active genes in samples. This correlation is weaker in normal samples and stronger in tumor samples.

The correlation is notable for the two genes with the highest frequencies in the T-panel, EPHA10 and UCN, as shown in Supplementary Figure 3. This means that the progression mechanisms or pathways labeled by these genes are activated along the evolution of many tumors.

Supplementary Figure 4 plots the mean number of active panel genes in normal samples and tumors. The correlation with the total number of active genes is statistically significant in both cases, with Pearson coefficients higher than 0.8. For tumors, the number of active genes in the panel may act as a rough indicator of staging. However, for normal samples it does not provide a practical index because the mean number of active genes is between 1 and 2.

S4. Molecular taxonomy of normal samples and tumors

The complete panels shown in sections 3.4 and 3.5 of the main manuscript provide an exhaustive taxonomy of normal samples and tumors. The limited knowledge about gene ontology and functions of genes in general, and in particular of panel genes prevent the interpretation of the clusters or groups of samples that follow from the panels. Thus, this section has an illustrative character only. A detailed characterization and validation requires additional work, which goes beyond the scope of the present paper.

Supplementary Figure 5 shows the dendrogram resulting from applying a Dice dissimilarity distance to activation patterns in the set of normal samples. Conventions are the same as in Figure 5, left panel, of the main manuscript. That is, genes preserve the same order and a black cell means that the gene is activated in that sample. Samples are organized along the *y* axis according to the obtained clusters. Using a threshold distance of 0.7, five groups and one isolated pattern (HSPA1L active) emerge. The interpretation is lacking, but the figure shows that we may construct a complete classification, covering the 100% of samples.

In the same way, Supplementary Figure 6 shows the corresponding dendrogram for tumors. Note that there are much more clusters or groups in tumors than in normal samples, revealing a richer structure, which is connected with the different entropies of the normal and tumor attractors.

S5. Comparison with traditional driver genes in PRAD

There are different molecular classifications of prostate tumors [60]. As an example, we briefly revisit the TCGA classification, which is essentially multi-omics but is labeled by a set of seven mutational driver genes [5], listed in Supplementary Table III. The classification algorithm was applied to an initial cohort of 333 samples, not the 499 tumor samples we are using in our study.

The resulting gene panel is incomplete, providing classification for only 76% of the samples. In Supplementary Table IV, we show a schematic representation of the TCGA classification, which illustrates the principle guiding the search for panel genes: their mutually exclusive character. In other words, when a gene is mutated in a sample, the other genes in the panel are not mutated.

In contrast, our classification is complete (100% sensitivity and 100% specificity within the training set), and the number of active panel genes serves as a rough indicator of the degree of progression.

Let us examine the TCGA panel genes in light of our scheme. Four of the genes—ERG, ETV1, ETV4, and FLI1—belong to the ETS family of transcription factors and show characteristic fusion patterns in PRAD. The other three genes—SPOP, FOXA1, and IDH1—exhibit characteristic mutations in PRAD. Notably, they consider fusion or mutation frequencies as low as 1%.

Recall that in our Fisher test, we adopt a threshold of 10% for T-activation frequency. Within these strict conditions of statistical significance, the seven-gene panel suggested by TCGA does not arise in our scheme. However, upon relaxing this threshold, they emerge from our analysis either as T-outside genes (as in the case of the four fusion genes and IDH1) or as NT-genes (as in the case of SPOP and FOXA1). Only under these relaxed conditions can we assess their role in tumorigenesis and tumor progression within our framework.

In general, the frequency of observed relevant mutations in PRAD is low [61]. In contrast, activations of T-genes are very common, reaching a maximum frequency of around 74% for the EPHA10 gene in the set of tumor samples. The histogram showing the number of T-genes in PRAD as a function of their activation frequencies is presented in Supplementary Figure 7. For example, there are more than 200 genes deregulated in 30% of tumor samples. In other tumors, such as LUAD and LIHC, activation frequencies are even higher. This illustrates another advantage of our scheme, based on T-gene expression activation: because our approach identifies T-genes with frequent and statistically significant activations across patients, it points to robust targets for therapeutic interventions.

References for Supplementary Material

[28] Xu M, Takanashi M, Oikawa K, et al. Identification of a novel role of Septin 10 in paclitaxel-resistance in cancers through a functional genomics screen. Cancer Sci. 2012;103(4):821-826. doi:10.1111/j.1349-7006.2012.02214.x

[29] Poüs C, Klipfel L, Baillet A. Cancer-related functions and subcellular localizations of septins. Front Cell Dev Biol. 2016;4:126. doi:10.3389/fcell.2016.00126

[30] Zhang Y, Zhang R, Liang F, Zhang L, Liang X. Identification of metabolism-associated prostate cancer subtypes and construction of a prognostic risk model. Front Oncol. 2020;10:598801. doi:10.3389/fonc.2020.598801

[31] Liu H, Jiang J, Tan Y, et al. Unraveling diethyl phthalate-induced prostate carcinogenesis: core targets revealed by integrated network toxicology, machine learning, and structural validation. Hum Genomics. 2025;19(1):18. doi:10.1186/s40246-025-00716-7

[32] Guo J, Cheng WP, Gu J, et al. Systemic delivery of therapeutic small interfering RNA using a pH-triggered amphiphilic poly-L-lysine nanocarrier to suppress prostate cancer growth in mice. Eur J Pharm Sci. 2012;45(5):521-532. doi:10.1016/j.ejps.2011.11.009

[33] Popiel A, Kobierzycki C, Dzięgiel P. The role of testin in human cancers. Pathol Oncol Res. 2019;25(4):1279-1284. doi:10.1007/s12253-018-0489-3

[34] Li H, Huang K, Gao L, et al. TES inhibits colorectal cancer progression through activation of p38. Oncotarget. 2016;7(29):45819-45832. doi:10.18632/oncotarget.10295

[35] Chêne L, Giroud C, Desgrandchamps F, et al. Extensive analysis of the 7q31 region in human prostate tumors supports TES as the best candidate tumor suppressor gene. Int J Cancer. 2004;111(5):798-804. doi:10.1002/ijc.20337

[36] Fladeby C, Gupta SN, Barois N, et al. Human PARM-1 is a novel mucin-like, androgen-regulated gene exhibiting proliferative effects in prostate cancer cells. Int J Cancer. 2008;122(6):1229-1235. doi:10.1002/ijc.23240

[37] Charfi C, Levros LC Jr, Edouard E, Rassart E. Characterization and identification of PARM-1 as a new potential oncogene. Mol Cancer. 2013;12:84. doi:10.1186/1476-4598-12-84

[38] Cornet AM, Hanon E, Reiter ER, et al. Prostatic androgen repressed message-1 (PARM-1) may play a role in prostatic cell immortalisation. Prostate. 2003;56(3):220-230. doi:10.1002/pros.10256

[39] Wang LY, Shi J, Wang MF, Liu YM, Guo HS. Characterization of RNA editing gene APOBEC3C as a candidate tumor suppressor in prostate cancer. Sci Rep. 2025;15(1):7123. doi:10.1038/s41598-025-90915-8

[40] Guo Y, Yu H, Li X, et al. APOBEC3B-driven mutations negatively regulated by P53 promote tumor progression and immunosuppressive microenvironment in prostate cancer. Mol Cell Biochem. 2025;480(3):1815-1828. doi:10.1007/s11010-024-05059-8

[41] Li X, Wang Y, Deng S, et al. Loss of SYNCRIP unleashes APOBEC-driven mutagenesis, tumor heterogeneity, and AR-targeted therapy resistance in prostate cancer. Cancer Cell. 2023;41(8):1427-1449.e12. doi:10.1016/j.ccell.2023.06.008

[42] Pan J, Wu Q, Zhao Y, et al. TMT1A inhibits lung adenocarcinoma progression by suppressing M2 macrophage polarization. PeerJ. 2023;11:e15799. doi:10.7717/peerj.15799

[43] Zhang D, Zou T, Liu Q, et al. Transcriptomic characterization revealed that METTL7A inhibits melanoma progression via the p53 signaling pathway and immunomodulatory pathway. [Journal Title]. [Year];[Volume]([Issue]):[Pages]. doi:[DOI]

[44] Li H, Ge Y, Wang Z, Liu Y, Wei P. Neurotransmitter release cycle-related genes predict the prognosis of lung adenocarcinoma. Medicine (Baltimore). 2022;101(40):e30937. doi:10.1097/MD.0000000000030937

[45] Sørensen KD, Wild PJ, Mortezavi A, et al. Genetic and epigenetic SLC18A2 silencing in prostate cancer is an independent adverse predictor of biochemical recurrence after radical prostatectomy. Clin Cancer Res. 2009;15(4):1400-1410. doi:10.1158/1078-0432.CCR-08-2204

[46] Haldrup C, Lynnerup AS, Storebjerg TM, et al. Large-scale evaluation of SLC18A2 in prostate cancer reveals diagnostic and prognostic biomarker potential at three molecular levels. Mol Oncol. 2016;10(4):615-625. doi:10.1016/j.molonc.2015.11.010

[47] Chauhan W, Sudharshan SJ, Kafle S, et al. SnoRNAs: exploring their implication in human diseases. Int J Mol Sci. 2024;25(13):7202. doi:10.3390/ijms25137202

[48] Lu B, Chen X, Liu X, et al. C/D box small nucleolar RNA SNORD104 promotes endometrial cancer by regulating the 2′-O-methylation of PARP1. J Transl Med. 2022;20(1):618. doi:10.1186/s12967-022-03815-w

[49] Scieglinska D, Piglowski W, Chekan M, et al. Differential expression of HSPA1 and HSPA2 proteins in human tissues; tissue microarray-based immunohistochemical study. Histochem Cell Biol. 2011;135(4):337-350. doi:10.1007/s00418-011-0799-5

[50] Hu L, Tong J, Tian D, Liu Q. The N6-methyladenosine modified EphA10 promotes prostate cancer progression by activating the ERK/AKT pathway. Biochem Genet. 2025;63(1):199-214. doi:10.1007/s10528-024-10839-6

[51] Wang A, Zhu J, Li Y, et al. Comprehensive analysis of Epha10 as a predictor of clinical prognosis and immune checkpoint therapy efficacy in non-small cell lung cancer. Sci Rep. 2024;14(1):15328. doi:10.1038/s41598-024-66209-2

[52] Zhao W, Liu L, Li X, Xu S. EphA10 drives tumor progression and immune evasion by regulating the MAPK/ERK cascade in lung adenocarcinoma. Int Immunopharmacol. 2022;113(Pt A):109322. doi:10.1016/j.intimp.2022.109322

[53] Kamada M, Ikeda K, Fujioka K, et al. Expression of mRNAs of urocortin and corticotropin-releasing factor receptors in malignant glioma cell lines. Anticancer Res. 2012;32(12):5299-5307.

[54] Mavridis C, Paspalaki E, Makrigiannakis A, Gravanis A. The corticotropin-releasing factor family in the urogenital system. Mol Med Rep. 2025;31(3):55. doi:10.3892/mmr.2024.13420

[55] Kikutake C, Yahara K. Identification of epigenetic biomarkers of lung adenocarcinoma through multi-omics data analysis. PLoS One. 2016;11(4):e0152918. doi:10.1371/journal.pone.0152918

[56] Hu L, Tong J, Tian D, Liu Q. The N6-methyladenosine modified EphA10 promotes prostate cancer progression by activating the ERK/AKT pathway. Biochem Genet. 2025;63(1):199-214. doi:10.1007/s10528-024-10839-6

[57] Sigorski D, Wesołowski W, Gruszecka A, et al. Neuropeptide Y and its receptors in prostate cancer: associations with cancer invasiveness and perineural spread. J Cancer Res Clin Oncol. 2023;149(9):5803-5821. doi:10.1007/s00432-022-04530-9

[58] Jiang YZ, Yu KD, Peng WT, et al. Enriched variations in TEKT4 and breast cancer resistance to paclitaxel. Nat Commun. 2014;5:3802. doi:10.1038/ncomms4802

[59] Galletti G, Matov A, Beltran H, et al. ERG induces taxane resistance in castration-resistant prostate cancer. Nat Commun. 2014;5:5548. doi:10.1038/ncomms6548

[60] Ge Q, Li J, Yang F, et al. Molecular classifications of prostate cancer: basis for individualized risk stratification and precision therapy. Ann Med. 2023;55:2279235.

[61] Wang G, Zhao D, Spring DJ, DePinho RA. Genetics and biology of prostate cancer. Genes Dev. 2018;32:1105-1140.

Captions of supplementary figures

Supplementary Figure 1. Active N-genes vs. distance to tumor attractor for LUAD and LIHC.

Analogous to Figure 4a. (a) LUAD: N-genes = 492, distance range ~ 170 - 210 units. (b) LIHC: N-genes = 581, distance range ~ 140 - 190 units. In both cases, the number of active N-genes decreases continuously as samples approach the transition region.

Supplementary Figure 2. Active T-genes vs. distance to normal attractor for LUAD and LIHC.

Analogous to Figure 4b. (a) LUAD: T-genes = 17037. (b) LIHC: T-genes = 17351. In both cases, the number of active T-genes increases continuously with distance from the normal attractor, with steeper slopes than in PRAD, consistent with larger attractor distances and more aggressive clinical behaviour.

Supplementary Figure 3. Mean T-activation of EPHA10 and UCN as a function of total T-gene activation in PRAD tumor samples.

Samples were grouped into contiguous bins according to total T-gene activation, and curves show sample-weighted three-bin running averages.

Supplementary Figure 4. Mean number of active panel genes as a function of total gene activation in PRAD samples.

Tumor and normal samples were grouped into contiguous bins according to total T- and N-gene activation, respectively. Curves show sample-weighted three-bin running averages for the eight-gene T-panel and eleven-gene N-panel.

Supplementary Figure 5. Dendrogram of normal samples based on N-panel activity patterns

Dice dissimilarity distance and complete linkage clustering. Using a threshold of 0.7, five groups and one isolated pattern (HSPA1L active) emerge. Samples are labeled by their total number of active N-genes. This represents the first molecular classification of normal tissues based on N-gene panels; clinical relevance requires validation.

Supplementary Figure 6. Dendrogram of tumor samples based on T-panel activity patterns

Dice dissimilarity distance and complete linkage clustering. Clusters correlate with total active T-gene counts, indicating that the panel captures the progression axis. Early (low active T-gene) and advanced (high active T-gene) tumors form distinct clusters.

Supplementary Figure 7. Histogram of T-gene deregulation frequencies in PRAD.

Number of T-genes (y-axis) versus frequency of activation (proportion of tumour samples, x-axis). Most T-genes have frequencies between 0.1 and 0.4. More than 200 genes are deregulated in 30% of tumour samples. EPHA10 shows the highest frequency (0.74).

Captions of supplementary tables

Supplementary Table I: Block sizes and frequencies for the five largest N-gene blocks in PRAD.

Supplementary Table II: Detailed annotations of N-panel and T-panel genes in the Reactome database and on the basis of recent literature.

Extended version of Table I, including gene names, categories, frequencies, block sizes, and detailed functional descriptions with literature references. For uncharacterized genes (ENSG00000224984, RPL26P23, ENSG00000271895) no functional information is available.

Supplementary Table III: TCGA driver panel genes and their frequencies.

Supplementary Table IV: TCGA classification schematic (mutually exclusive pattern).

Figures and Tables

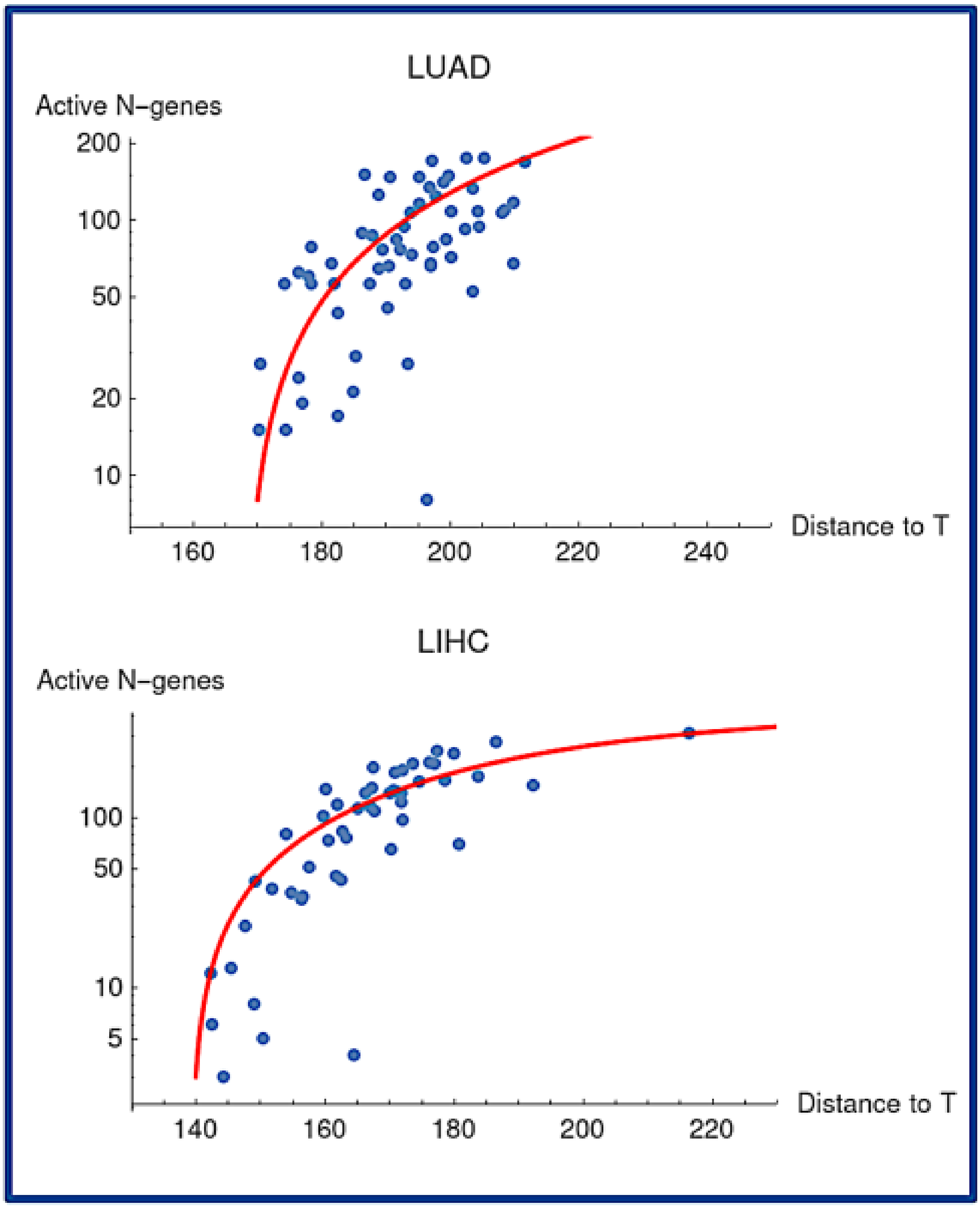

Supplementary Figure 1.

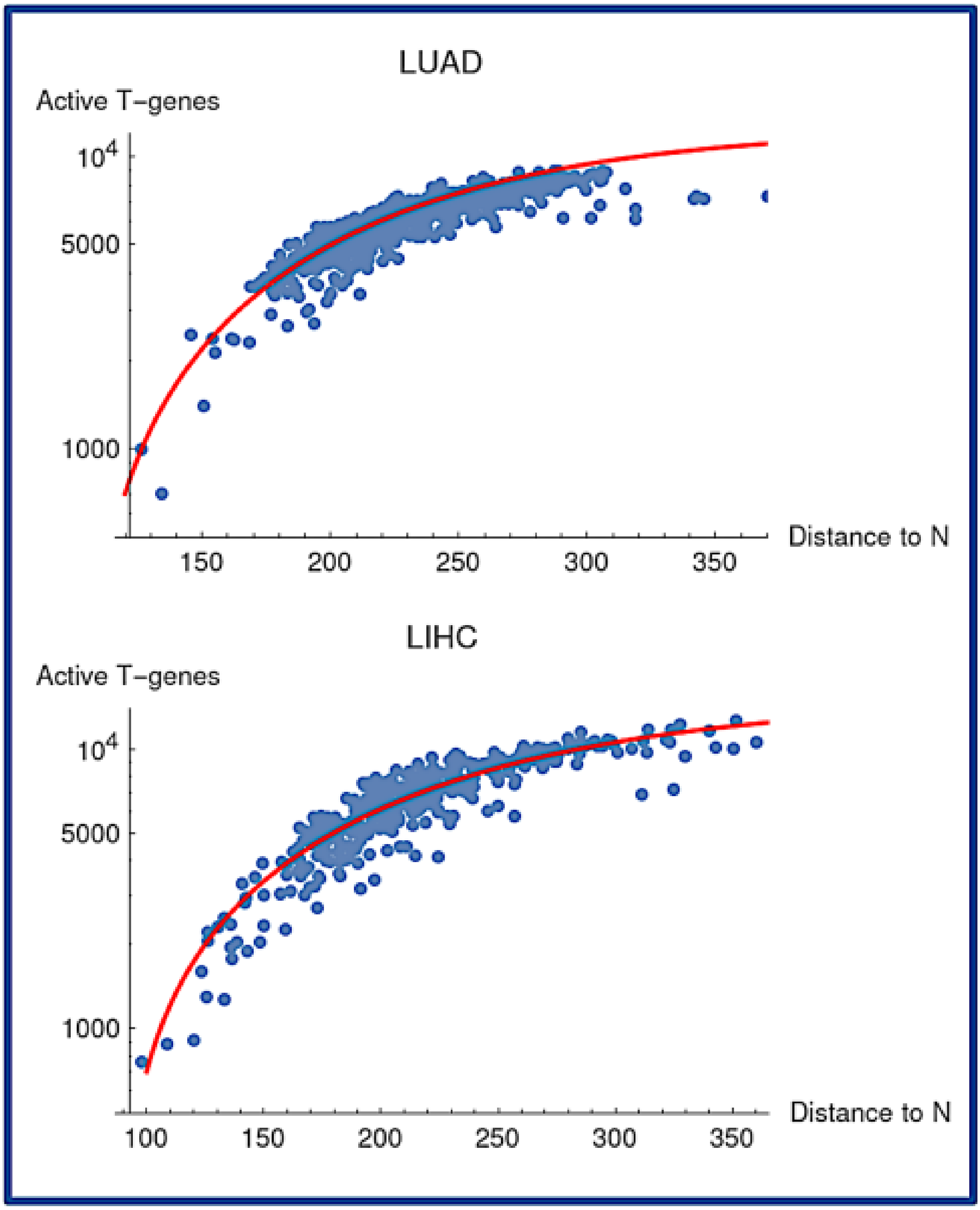

Supplementary Figure 2.

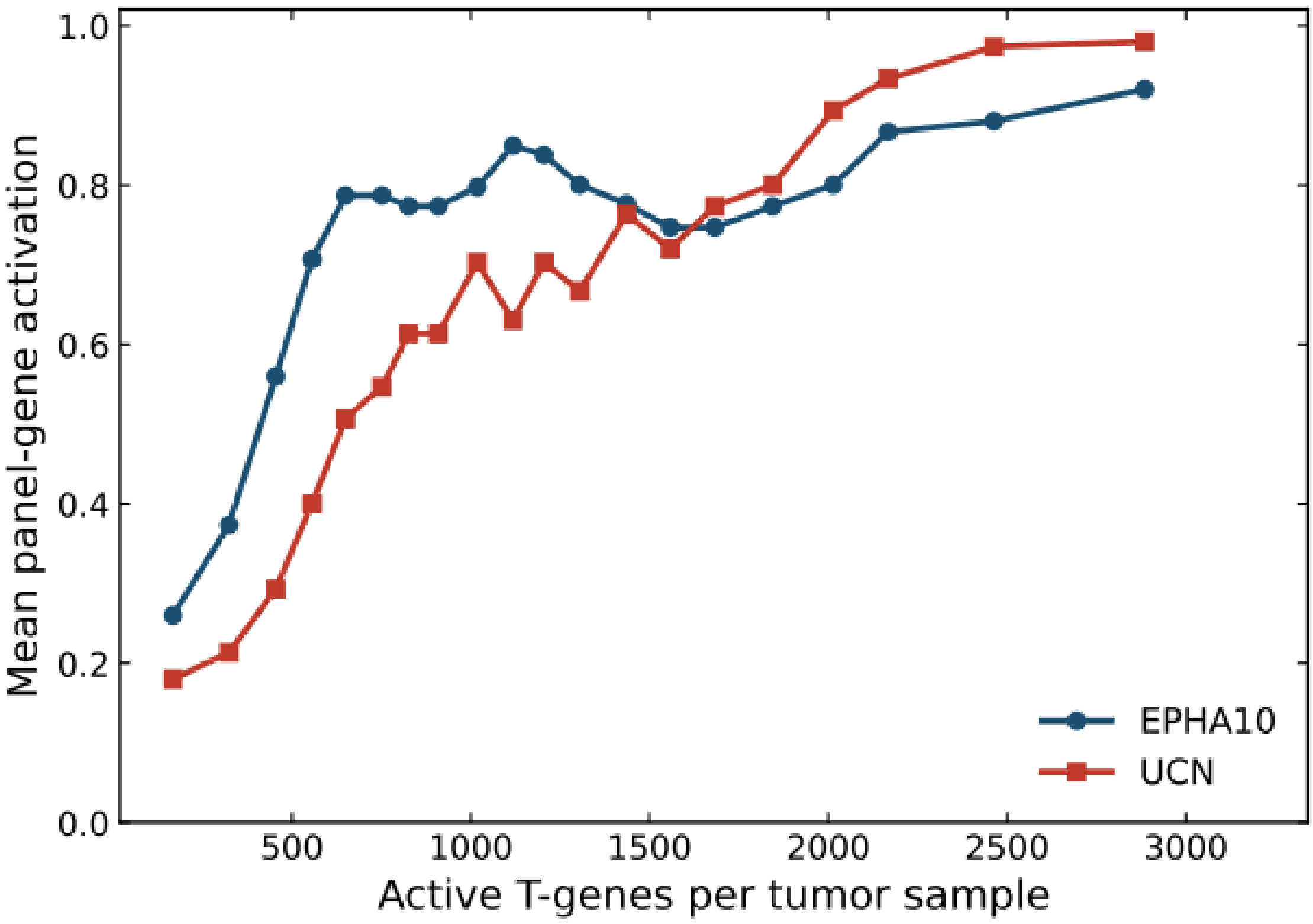

Supplementary Figure 3

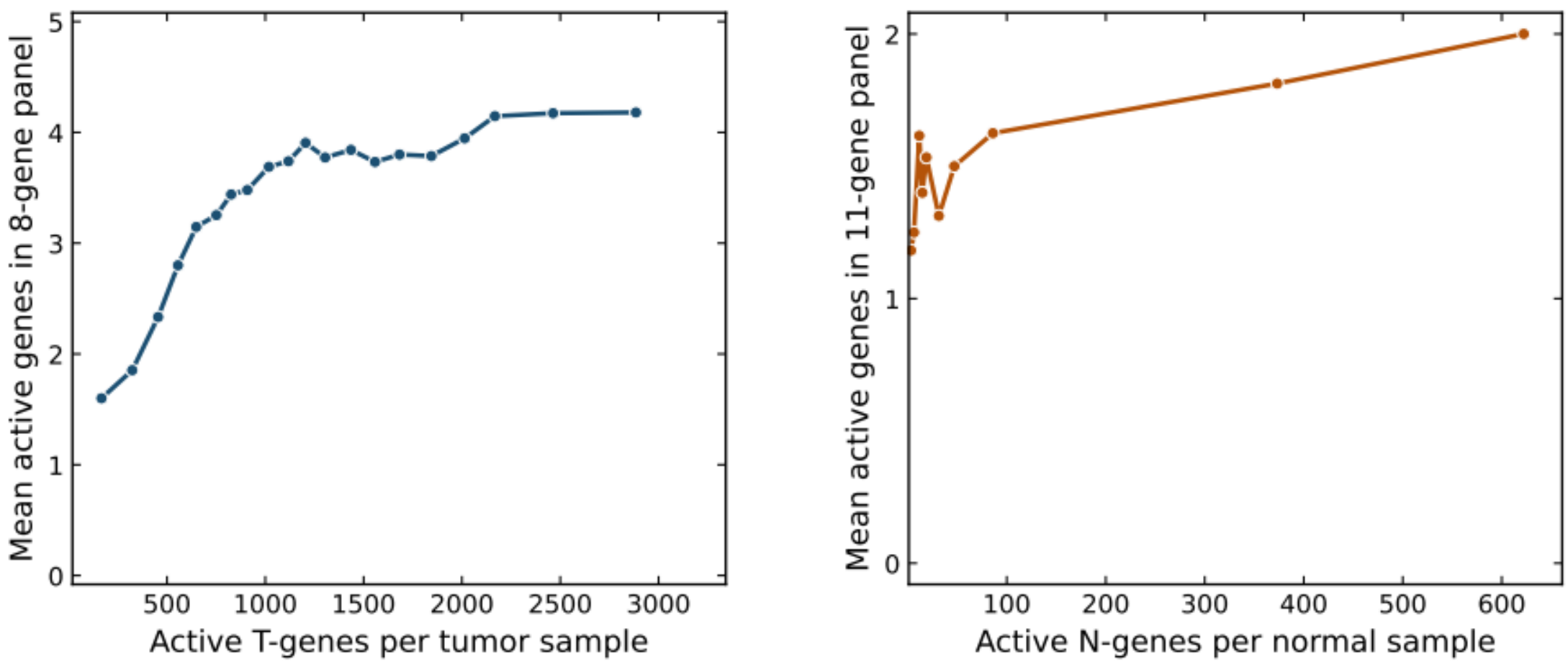

Supplementary Figure 4

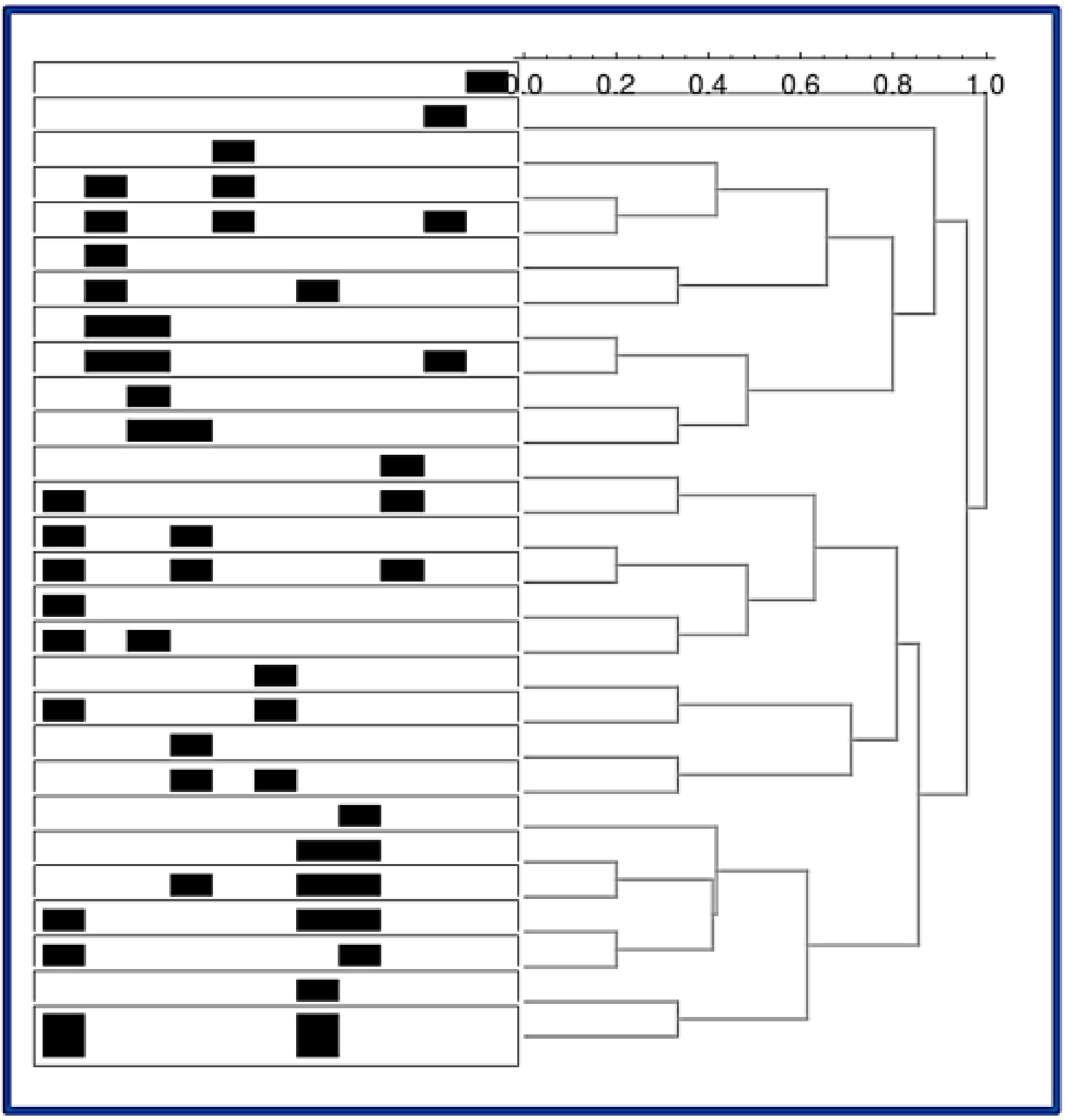

Supplementary Figure 5

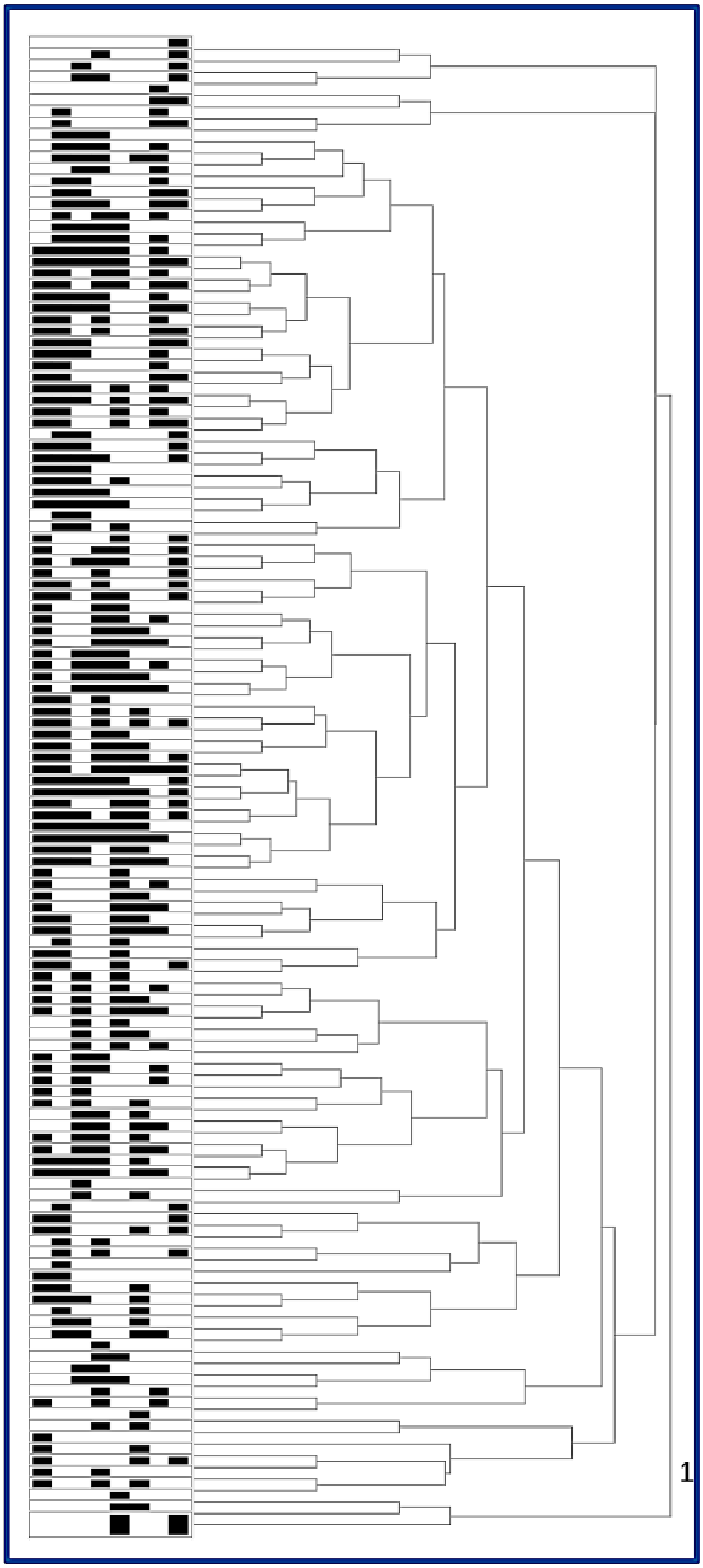

Supplementary Figure 6

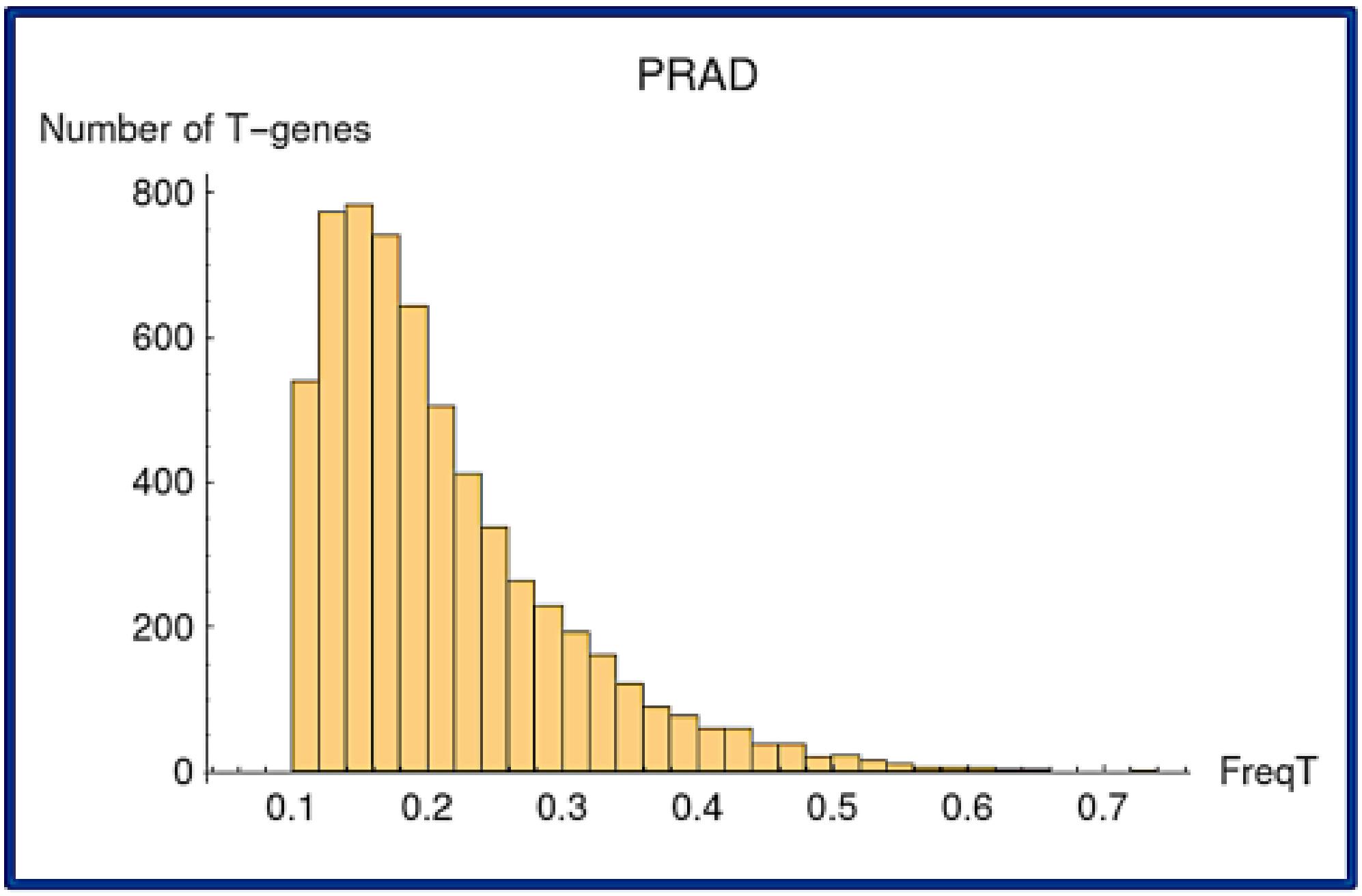

Supplementary Fig. 7.

Block N-genes	Frequency in normal samples	Number of genes in the block
Block 1	0.13	216 genes
Block 2	0.11	34 genes
Block 3	0.05	28 genes
Block 4	0.11	27 genes
Block 5	0.15	20 genes

Supplementary Table I

N-genes in the perfect panel	Pathways in the Reactome database [27]	Reported protective role based on recent literature
SEPTIN10	Non annotated	Ensures accurate cell division (through its interaction with BUB3) and facilitates the death of cells with damaged microtubules [28]. SEPTIN10 acts as a dual caretaker and gatekeeper. The protective function is likely dependent on the specific protein environment of a healthy prostate cell. If that environment changes—for example, if a tumor does form—SEPTIN10's role could be subverted to support cancer progression [29], as seen in other tissues like the liver.
CA14	R-HSA-1475029 Reversible hydration of carbon dioxide<br>R-HSA-1430728 Metabolism	Regulates pH balance and, as part of a normal metabolic program, helps maintain a cellular environment that is hostile to cancer initiation [30,31]. Loss of CA14 expression is a key step in prostate cancer development and progression [32].
TES	R-HSA-5683826 Surfactant metabolism<br>R-HSA-392499 Metabolism of proteins	Its protein product helps maintain normal cell structure and adhesion [33]. It can activate stress-response pathways like p38 to eliminate potentially dangerous cells [34]. It is a key "gatekeeper" in the prostate epithelium [35].
PARM1	Non annotated	Appears to function as an androgen-repressed, pro-survival factor. Its primary role in prostate cells may be to help them survive periods of low androgen. This could be seen as a protective mechanism for the cell, but it's one that cancer cells can exploit to their advantage, promoting immortalization and proliferation [36,37,38].
APOBEC3C	R-HSA-75094 Formation of the Editosome<br>R-HSA-72200 mRNA Editing: C to U Conversion<br>R-HSA-75072 mRNA Editing<br>R-HSA-8953854 Metabolism of RNA	Functions as a tumor suppressor in prostate cells [39]. Its expression maintains normal growth control, suppressing proliferation, migration, and invasion . Its loss is an early step that removes a barrier to cancer development. It is one member of a family of enzymes. While it protects, other family members (like APOBEC3B) are potent mutators that drive cancer progression, heterogeneity, and therapy resistance [40] when they are "unleashed" by the loss of regulatory proteins like SYNCRIP [41].
ENSG00000224984	Non annotated	LncRNA. Uncharacterized.
TMT1A	R-HSA-6798695 Neutrophil degranulation<br>R-HSA-168249 Innate Immune System	Functions as a multi-faceted tumor suppressor by: 1) Directly inhibiting cancer cell proliferation and migration (potentially via p53) , 2) Shaping a favorable immune microenvironment by suppressing tumor-promoting M2 macrophages and enhancing T cell activity, and 3) Potentially regulating cell-cell communication through its RNA methyltransferase activity [42]. The significant downregulation of TMT1A in prostate adenocarcinoma tissues is entirely consistent with it being a tumor suppressor whose loss is selected for during cancer development. This mirrors its behavior in lung cancer and melanoma [43].
SLC18A2	R-HSA-181429 Serotonin Neurotransmitter Release Cycle<br>R-HSA-212676 Dopamine Neurotransmitter Release Cycle<br>R-HSA-181430 Norepinephrine Neurotransmitter Release Cycle<br>R-HSA-112310 Neurotransmitter release cycle<br>R-HSA-112315 Transmission across Chemical Synapses<br>R-HSA-112316	Essential for normal neuroendocrine cell function in the prostate. By regulating the release of signaling molecules, it helps maintain tissue homeostasis and suppresses aberrant epithelial proliferation [44]. It acts as a gatekeeper of the normal prostate microenvironment. It is specifically and comprehensively silenced in prostate cancer through early, concurrent genetic loss (LOH) and epigenetic hypermethylation [45,46]. This silencing removes its protective influence, creating a permissive environment for malignant transformation.

	Neuronal System	
SNORD104	Non annotated	There is not current evidence for a protective effect in prostate, although other SnoRNAs have been shown to promote proliferation and migration in prostate cancer [47]. The emerging picture from endometrial cancer suggests it is an oncogene that could potentially drive prostate cancer progression through PARP1 regulation [48].
RNA5SP342	Non annotated	Ribosomal pseudogene. Uncharacterized.
HSPA1L	R-HSA-3371568<br>Attenuation phase<br>R-HSA-3371571<br>HSF1-dependent transactivation<br>R-HSA-3371453<br>Regulation of HSF1-mediated heat shock response<br>R-HSA-3371511<br>HSF1 activation<br>R-HSA-3371556<br>Cellular response to heat stress<br>R-HSA-2262752<br>Cellular responses to stress<br>R-HSA-8953897<br>Cellular responses to stimuli<br>R-HSA-3371497<br>HSP90 chaperone cycle for steroid hormone receptors (SHR)<br>in the presence of ligand<br>R-HSA-9833482<br>PKR-mediated signaling<br>R-HSA-1169410<br>Antimicrobial mechanism of IFN-stimulated genes<br>R-HSA-913531<br>Interferon Signaling<br>R-HSA-1280215<br>Cytokine Signaling in Immune system	Expressed in normal prostate epithelium as part of the cellular stress response machinery [49]. It helps maintain protein homeostasis and cell survival under stress. More experimental evidence is needed.
**T-genes in the perfect panel**	**Pathways in the Reactome database [27]**	**Reported oncogenic role based on recent literature**
EPHA10	R-HSA-3928663<br>EPHA-mediated growth cone collapse<br>R-HSA-3928665<br>EPH-ephrin mediated repulsion of cells<br>R-HSA-2682334<br>EPH-Ephrin signaling<br>R-HSA-422475<br>Axon guidance<br>R-HSA-9675108<br>Nervous system development<br>R-HSA-1266738<br>Developmental Biology	Oncogenic driver; m6A-methylation regulated by RBM15B; activates ERK/AKT pathway [50]; potential immune evasion [51,52].
UCN	R-HSA-422085<br>Synthesis, secretion, and deacylation of Ghrelin<br>R-HSA-2980736<br>Peptide hormone metabolism<br>R-HSA-373080<br>Class B/2 (Secretin family receptors)	UCN-CRFR2 signaling may act as an endogenous inhibitor of angiogenesis and proliferation; receptor loss (CRFR2) may disrupt tumor suppression [53,54].

	R-HSA-500792<br>GPCR ligand binding<br>R-HSA-372790<br>Signaling by GPCR<br>R-HSA-392499<br>Metabolism of proteins<br>R-HSA-162582<br>Signal Transduction	
TMEM86A	R-HSA-1482788<br>Acyl chain remodelling of PC<br>R-HSA-1483206<br>Glycerophospholipid biosynthesis<br>R-HSA-1483257<br>Phospholipid metabolism<br>R-HSA-556833<br>Metabolism of lipids<br>R-HSA-1430728<br>Metabolism	Transmembrane protein; epigenetically regulated (H3K27me3) [55]; cited in prostate cancer context.
RPL26P23	Non annotated	Pseudogene (likely). Uncharacterized
RBM15B	Non annotated	Oncogenic m6A writer; Methylates EPHA10, PCNA mRNAs; stabilizes transcripts; promotes proliferation [56].
NPY4R2	Non annotated	A member of the neuropeptide Y receptor family. Other neuropeptide receptors (e.g., NPY1R, NPY5R) have been implicated in prostate cancer progression, neuroendocrine differentiation, and bone metastasis. By analogy, NPY4R2 could play a role, but no data supports this speculation [57].
TEKT4	Non annotated	Chemotherapy resistance; Microtubule-associated protein; mutations alter taxane binding [58]; linked to ERG fusion status [59].
ENSG00000271895	Non annotated	Uncharacterized

Supplementary Table II.

Gene	Mutation or Fusion Freq.	Classif.	Freq(+)	Freq(-)
ERG (fusion)	0.46	T-outside?	0.23	0.01
ETV1 (fusion)	0.08	T-outside?	0.08	0.08
ETV4 (fusion)	0.04	T-outside?	0.05	0.05
FLI1 (fusion)	0.01	T-outside?	0.01	0.06
SPOP (mutation)	0.11	NT-gene?	0.154 (N)	0.042 (T)
FOXA1 (mutation)	0.03	NT-gene?	0.046 (T)	0.154 (N)
IDH1 (mutation)	0.01	T-outside?	0.04	0.02

Supplementary Table III.

Samples →	(46%)	(8%)	(4%)	(1%)	(11%)	(3%)	(1%)	(26%)
ERG	1	0	0	0	0	0	0	0
ETV1	0	1	0	0	0	0	0	0
ETV4	0	0	1	0	0	0	0	0
FLI1	0	0	0	1	0	0	0	0
SPOP	0	0	0	0	1	0	0	0
FOXA1	0	0	0	0	0	1	0	0
IDH1	0	0	0	0	0	0	1	0

Supplementary Table IV.
